# Treatment with orforglipron, an oral glucagon like peptide-1 receptor agonist, is associated with improvements of CV risk biomarkers in participants with type 2 diabetes or obesity without diabetes

**DOI:** 10.1186/s12933-025-02781-x

**Published:** 2025-06-06

**Authors:** Sean Wharton, Julio Rosenstock, Manige Konige, Yanzhu Lin, Kevin Duffin, Jonathan Wilson, Hiya Banerjee, Valentina Pirro, Christof Kazda, Kieren Mather

**Affiliations:** 1 McMaster University, York University, and Wharton Weight Management Clinic, Toronto, Canada; 2 Velocity Clinical Research at Medical City, Dallas, TX USA; 3 Eli Lilly and Company, Indianapolis, IN USA

**Keywords:** GLP-1, Orforglipron, Oral, Diabetes, Obesity

## Abstract

**Background:**

Orforglipron, a novel oral, non-peptide glucagon like peptide-1 (GLP-1) receptor agonist, has demonstrated efficacy in improving body weight reduction and glycemic control. However, its potential benefits in improving cardiovascular (CV) risk factors have yet to be determined. We assessed the effect of orforglipron in participants with type 2 diabetes (T2D) and/or overweight or obesity on blood pressure, lipid, and inflammatory biomarkers associated with risk for major adverse cardiovascular events.

**Methods:**

Using data from participants with available samples from Phase 2 trials of orforglipron in participants with T2D (N = 361) or with overweight or obesity without diabetes mellitus (N = 234), we performed an exploratory analysis of changes in CV risk markers. For the T2D study, participants mean age 59 years, 40% were assigned female at birth with a mean HbA_1c_ of 8.1% and mean BMI of 35.3 kg/m^2^; they received once daily orforglipron doses (3, 12, 24, 36, or 45 mg) or once weekly subcutaneous dulaglutide 1.5 mg, or placebo. In the obesity study, participants had a mean age 54 years, 60% were assigned female at birth, and mean BMI was 37.9 kg/m^2^; they received once daily orforglipron (12, 24, 36, or 45 mg) or placebo. The change from baseline at 26 weeks (T2D study) or 36 weeks (obesity study) in blood pressure, lipids (cholesterol, triglycerides, Apolipoprotein B (ApoB), Apolipoprotein C3 (ApoC3), N-terminal pro-b-type natriuretic peptide (NT-pro-BNP), and inflammatory biomarkers (high-sensitivity C-reactive protein (hsCRP), interleukin-6 (IL-6)) were assessed.

**Results:**

Significant placebo-adjusted decreases from baseline in blood pressure, low-density lipoprotein (LDL) cholesterol, triglycerides, ApoB, ApoC3, and hsCRP were observed following orforglipron treatment in participants with T2D and/or overweight or obesity. In both studies, improvements in blood pressure, lipid parameters, and most of the evaluated biomarkers were of similar magnitude after treatment with 12 mg orforglipron as with 24, 36, and 45 mg.

**Conclusion:**

Orforglipron treatment was associated with beneficial changes in CV risk markers in participants with T2D and in participants with overweight/obesity without T2D. (Clinicaltrials.gov: NCT05048719, NCT05051579).

**Graphical abstract:**

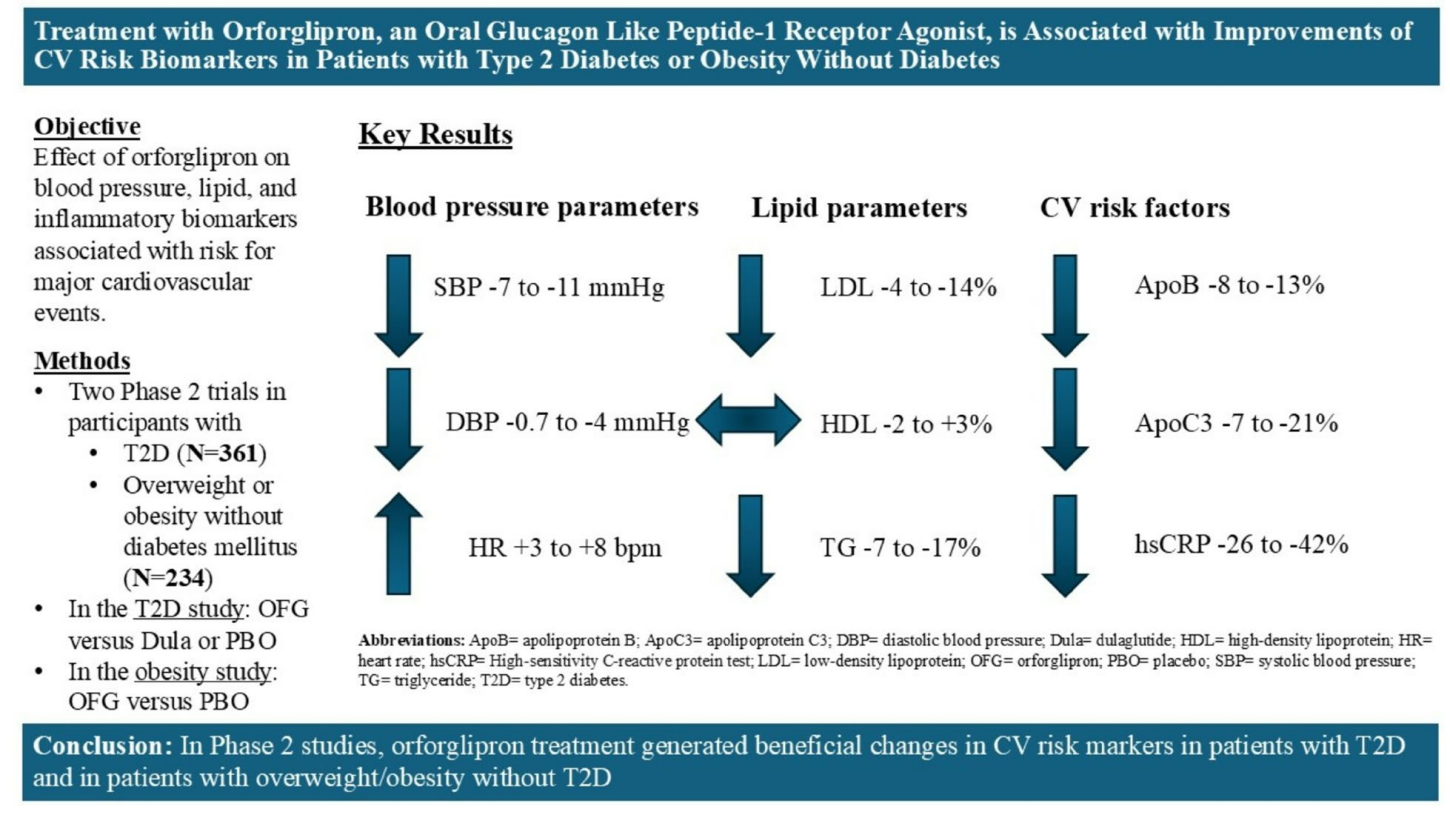

**Supplementary Information:**

The online version contains supplementary material available at 10.1186/s12933-025-02781-x.

## Research awareness

**What is currently known about this topic?**

- Cardiovascular (CV) disease remains a significant threat in people with obesity and type 2 diabetes (T2D), with a two to three times higher incidence of CVD compared to people without diabetes or obesity. Injected incretins have been successful in reducing CV risk in these populations.

**What is the key research question?**

- Does the small molecule non-peptide GLP-1 receptor agonist orforglipron improve CV risk biomarkers in participants with T2D and participants with obesity without diabetes?

**What is new?**

- In the Phase 2 studies of orforglipron, we observed significant reductions in blood pressure, lipid levels, and inflammatory markers, highlighting the potential of orforglipron to improve CV risk factors in these at-risk populations.

**How might this study influence clinical practice?**

- These effects are consistent with the observed cardiovascular benefits of peptide-based GLP-1 receptor agonists and suggest the potential for novel small molecule receptor agonists to provide similar benefits while providing a needle-free alternative treatment option.

## Introduction

Cardiovascular disease (CVD) remains a significant threat to people with obesity and type 2 diabetes (T2D), with a two to three times higher incidence compared to people without diabetes or obesity [1, 2]. In addition to improving glucose levels and weight, subcutaneous glucagon-like peptide-1 receptor agonists (GLP-1 RAs) have produced meaningful reductions in major adverse cardiovascular events (MACE) [3]. For example, in participants with type 2 diabetes, treatment with dulaglutide reduced the risk of CV death, myocardial infarction, or non-fatal stroke by 12%, while liraglutide produced a 13% reduction an analogous 3-point composite MACE [4, 5]. The SELECT trial with injected semaglutide demonstrated a 20% reduction in the frequency of 3-point MACE outcome in people with preexisting CVD and overweight/obesity without T2D [6], and injected semaglutide has been shown to reduce 3-point MACE outcomes by 26% in patients with pre-existing CVD and T2D [7]. Orally delivered semaglutide produces improvements in cardiovascular risk factors [8, 9], and has demonstrated potential to reduce the risk of 3-point MACE in participants with type 2 diabetes with established CVD [10].

Biomarkers such as elevated blood pressure, lipid levels, and inflammatory markers are closely associated with CVD risk, and treatments that reduce these markers can reduce CVD risk in populations with and without diabetes [11–13]. Dulaglutide, liraglutide, and semaglutide effectively reduce LDL cholesterol, high-sensitivity C-reactive protein (hs-CRP), and triglycerides, and reduce cardiovascular risk. For example, in participants with type 2 diabetes liraglutide reduced low-density lipoprotein (LDL) cholesterol by approximately 10%, triglycerides by around 16%, and hs-CRP by about 35%, in association with a 13% reduction in MACE events [14, 15]. In participants with overweight/obesity with and without type 2 diabetes, injected semaglutide similarly reduced LDL by up to 7%, triglycerides by up to 18%, and c-reactive protein by up to 25% in association with reductions in cardiovascular outcomes [9, 16]. These observations underscore the relevance of using these biomarkers to assess potential beneficial effects on cardiovascular risk with GLP-1 receptor agonism [17].

More recently investigated biomarkers of cardiovascular risk include apolipoprotein B (ApoB), apolipoprotein C3 (ApoC3), N-terminal pro-b-type natriuretic peptide (NT-proBNP), and interleukin-6 (IL-6). ApoB and ApoC3 are components of circulating lipoprotein particles, and have been linked to atherosclerosis and treatment response [18–20]. NT-proBNP is an established indicator of heart failure and cardiovascular stress with dynamic response to disease state and treatment [21–23], while IL-6 is a cytokine that is upstream of C-reactive protein and provides a complementary marker of systemic inflammation, which has been successfully targeted in the treatment of CVD [24]. GLP-1 receptor agonists such as semaglutide and liraglutide have shown reductions in ApoB, ApoC3, NT-proBNP, and IL-6 levels. These biomarkers may therefore provide information about treatment effects on CV risk that supplement the information provided by traditional markers.

Orforglipron (OFG), an oral, once daily, non-peptide GLP-1 receptor agonist, demonstrated effective weight loss of up to − 10.1 kg at 26 weeks in T2D, and up to − 15.4 kg at 36 weeks in participants with obesity without T2D [25, 26]. This molecule also demonstrated improvement in HbA_1c_ (glycated haemoglobin) with reductions of up to − 2.1% at 26 weeks in participants with T2D in Phase 2 studies [25, 26]. Here we explored the treatment effects on cardiovascular risk factors in people with T2D and in people with overweight/or obesity without diabetes.

## Methods

### Trial designs and participants

The present analysis draws data from two completed Phase 2 multicenter studies with orforglipron [25, 26]. The T2D study was a randomized clinical trial (RCT) in which participants were assigned to placebo, once weekly dulaglutide 1.5 mg, or once daily orforglipron (3, 12, 24, 36, or 45 mg) for 26 weeks (NCT05048719). Randomization was stratified by country and HbA_1c_ stratum (above or below 8.0%) at their screening visit. Key eligibility criteria included adults with T2D and HbA_1c_ of 7.0–10.5%, with or without a stable dose of metformin for at least 3 months, a BMI of 23 kg/m^2^ or more, and a stable body weight (≤ 5% bodyweight gain or loss) for 3 months before randomization withe lifestyle and dietary measures for diabetes treatment. A second trial was conducted in adults with obesity (body mass index (BMI) ≥ 30 kg/m^2^), or with overweight (BMI 27 to 30 kg/m^2^) plus at least one weight-related complication, excluding diabetes (NCT05051579). Participants were randomly assigned to receive once daily orforglipron (12, 24, 36, or 45 mg) or placebo for 36 weeks as an adjunct to lifestyle intervention that included education regarding healthy eating and exercise provided by trial personnel. Randomization was stratified by baseline BMI (above or below 35 kg/m^2^ at the screening visit and by sex. Further details of the clinical trial protocols, study populations, statistical analysis plans, and study outcomes have been published [25, 26]. The trials adhered to the principles of the Declaration of Helsinki and received approval from an independent ethics committee or institutional review board at each participating site. Participants provided informed consent for study participation.

### Procedures

In the T2D study, dose escalation was performed at different weekly intervals in all treatment groups, lasting up to 16 weeks; two different dose escalation approaches were evaluated for the 36 mg and 45 mg cohorts. Similarly, in the obesity trial, dose escalation was employed in all cohorts including evaluation of two different approaches in the orforglipron 36 and 45 mg dose cohorts [25, 26].

### Outcomes

In order to assess treatment effects on cardiovascular risk, the current analyses expanded upon the evaluation of changes in blood pressure and lipid parameters that have been previously reported [25, 26], and added evaluation of additional biomarkers of CV risk (ApoB, ApoC3, NT-pro-BNP, hsCRP and IL-6) in participants who received placebo, dulaglutide, or orforglipron during the T2D and obesity trials. Blood pressure was measured using automated blood pressure cuffs in duplicate. Traditional lipids (total cholesterol, LDL cholesterol, high-density lipoprotein (HDL) cholesterol, and triglycerides) were measured at a central laboratory using an enzymatic method. For the T2D trial, ApoB, and ApoC3 were measured using serum and plasma samples. For the obesity trial, ApoB and ApoC3 were measured at Lilly Research Laboratories using a validated liquid chromatography-tandem mass spectrometry (LC–MS/MS) assay. Briefly, matching synthetic isotope-labeled apolipoprotein peptides were spiked into plasma samples to minimize analytical variability and normalize instrument response. Plasma apolipoproteins were then trypsin digested through an optimized procedure and peptides were quantified by LC–MS/MS in multiple reaction monitoring mode. Concentrations were calculated using reference serum calibrators. For both trials, hsCRP (Roche, Indianapolis, IN) and IL-6 (MesoScalse Discovery, Rockville, MD) were measured at Lilly Research Laboratories.

### Statistical analysis

This analysis set included all randomized participants with at least one dose of study drug, excluding data after the initiation of glycemic rescue medication (T2D study) or after permanent discontinuation of the study drug (both studies). For the 36 and 45 mg treatment groups, data from the two dose-escalation cohorts were pooled. Data were analyzed up to 26 weeks of treatment in the T2D trial and 36 weeks of treatment in the obesity trial.

Baseline measures were compared across dose groups using analysis of variance. The change from baseline over time was analyzed using mixed model repeated measures (where multiple post-baseline values were available) or analysis of covariance (where only one post-baseline value was available). The mixed model for both studies included treatment, visit, and their interaction as fixed effects. Treatment effects compared to placebo were tested at a two-sided significance level of *p* < 0.05, with two-sided 95% confidence intervals calculated. Statistical calculations were performed using SAS version 8.2. As these were exploratory analyses, no adjustments for multiple comparisons were made.

### Role of the funding source

The sponsor (Eli Lilly) was involved in study design, data collection, data analysis, data interpretation and writing of the report.

## Results

### Study participants

From September 2021 through September 2022, 383 participants were enrolled and randomly assigned to a treatment group in the T2D study; 361 (94%) participants had evaluable biomarker data for the current analyses. These participants received orforglipron 3 mg (*N* = 47), 12 mg (*N* = 50), 24 mg (*N* = 42), 36 mg (*N* = 59), 45 mg (*N* = 59), dulaglutide 1.5 mg (*N* = 50), or placebo (*N* = 54). Between September 2021 and late November 2022, 272 participants were randomized in the obesity study; 234 (86%) had evaluable data. These participants received orforglipron 12 mg (*N* = 43), 24 mg (*N* = 43), 36 mg (*N* = 49), 45 mg (*N* = 52), or placebo (*N* = 47). As previously reported, in these trials gastrointestinal events were the most common adverse effects in patients receiving orforglipron, consistent with the GLP-1 receptor agonist class [25, 26].

### Baseline demographics and characteristics

Baseline demographics and characteristics were well balanced across the treatment groups for the evaluable subgroups from both trials as shown in Tables 1 and 2. In the T2D trial, the mean age was 59 years; 40% were assigned female at birth and the population had mean HbA_1c_ of 8.1%, BMI of 35.3 kg/m^2^, (Table 1). In the obesity trial, the mean age was 54 years; 60% were assigned female at birth, and the population had mean BMI of 37.9 kg/m^2^ (Table 2).

**Table 1:** Baseline demographics and clinical variables for evaluable participants in T2D study

	Placebo (*N* = 54)	Orforglipron					Dulaglutide (*N* = 50)	Overall (*N* = 361)
		3 mg (*N* = 47)	12 mg (*N* = 50)	24 mg (*N* = 42)	36 mg (*N* = 59)	45 mg (*N* = 59)		
Age, years	58.8 (8.9)	58.7 (9.6)	57.1 (9.1)	60.1 (9.3)	59.4 (9.1)	58.5 (9.5)	58.8 (10.2)	58.7 (9.3)
Assigned female at birth, n (%)	27 (50.0)	22 (46.8)	18 (36.0)	13 (31.0)	25 (42.4)	21 (35.6)	20 (40.0)	146 (40.4)
Race, n (%)								
American Indian or Alaskan native	1 (1.9)	0	1 (2.0)	1 (2.4)	0	1 (1.7)	0	4 (1.1)
Asian	0	1 (2.1)	1 (2.0)	1 (2.4)	1 (1.7)	0	1 (2.0)	5 (1.4)
Black or African American	4 (7.4)	2 (4.3)	4 (8.0)	1 (2.4)	0	5 (8.5)	4 (8.0)	20 (5.5)
White	49 (90.7)	43 (91.5)	44.0 (88.0)	39 (92.9)	56 (94.9)	53 (89.8)	44 (88.0)	328 (90.9)
Multiple	0	1 (2.1)	0	0	2 (3.4)	0	1 (2.0)	4 (1.1)
Weight, kg	102.2 (18.9)	98.7 (25.4)	100.4 (17.6)	100.5 (22.0)	99.0 (17.8)	105.0 (25.6)	98.8 (22.1)	100.8 (21.5)
BMI, kg/m^2^	35.8 (6.2)	34.8 (7.5)	35.2 (6.3)	34.2 (7.9)	34.6 (5.5)	36.4 (7.0)	35.4 (8.0)	35.3 (6.9)
Waist circumference, cm	115.2 (12.4)	112.4 (18.1)	114.9 (11.2)	114.2 (15.0)	112.2 (12.9)	116.0 (17.0)	114.0 (16.4)	114.1 (14.8)
HbA_1c_, %	8.0 (0.8)	8.0 (0.8)	8.3 (0.9)	8.1 (0.9)	8.0 (0.7)	8.1 (0.9)	8.0 (0.7)	8.1 (0.8)
Fasting serum glucose, mg/dL	170.0 (40.4)	164.1 (41.5)	171.5 (43.5)	171.3 (43.7)	156.7 (28.2)	165.8 (35.4)	167.6 (38.0)	166.4 (38.6)
Waist to height ratio	0.7 (0.1)	0.7 (0.1)	0.7 (0.1)	0.7 (0.1)	0.7 (0.1)	0.7 (0.1)	0.7 (0.1)	0.7 (0.1)

Data presented are mean (SD) and n (%) unless otherwise noted. BMI = Body mass index; HbA_1c_ = glycated hemoglobin; N = number of randomized participants; n = number, SD = standard deviation

**Table 2:** Baseline demographics and clinical variables for evaluable participants in the obesity study

	Placebo (*N* = 47)	Orforglipron				Overall (*N* = 234)
		12 mg (*N* = 43)	24 mg (*N* = 43)	36 mg (*N* = 49)	45 mg (*N* = 52)	
Age, years	54.3 (8.9)	49.6 (10.7)	57.7 (9.2)	55.7 (11.8)	52.0 (11.0)	53.8 (10.9)
Assigned female at birth, n (%)	28 (59.6)	27 (62.8)	25 (58.1)	31 (63.3)	30 (57.7)	141 (60.3)
Race, n (%)						
American Indian or Alaskan native	0	0	1 (2.3)	0	0	1 (0.4)
Asian	2 (4.3)	0	0	0	0	2 (0.9)
Black or African American	1 (2.1)	3 (7.0)	5 (11.6)	7 (14.3)	1 (1.9)	17 (7.3)
White	42 (89.4)	40 (93.0)	37 (86.0)	42 (85.7)	50 (96.2)	211 (90.2)
Multiple	2 (4.3)	0	0	0	0	2 (0.9)
Body weight, kg	106.2 (22.3)	108.1 (26.7)	113.6 (32.0)	109.0 (27.3)	107.0 (21.3)	108.7 (25.9)
BMI, kg/m^2^	37.5 (6.0)	38.0 (8.1)	38.7 (8.2)	38.1 (6.7)	37.5 (6.2)	37.9 (7.0)
Waist circumference, cm	114.8 (14.3)	114.8 (17.5)	120.9 (20.6)	117.2 (16.0)	116.3 (13.2)	116.8 (16.4)
HbA_1c_, %	5.6 (0.4)	5.6 (0.4)	5.8 (0.3)	5.6 (0.4)	5.6 (0.3)	5.6 (0.4)
Fasting serum glucose, mg/dL	96.9 (10.4)	93.8 (10.2)	97.8 (11.3)	97.8 (12.7)	94.7 (9.9)	96.2 (11.0)
Waist to height ratio	0.7 (0.1)	0.7 (0.1)	0.7 (0.1)	0.7 (0.1)	0.7 (0.1)	0.7 (0.1)

Data presented are mean (SD) and n (%) unless otherwise noted. BMI = Body mass index; HbA_1c_ = glycated hemoglobin; N = number of randomized participants; n = number, SD = standard deviation

### Change from baseline in blood pressure

**Fig. 1:**
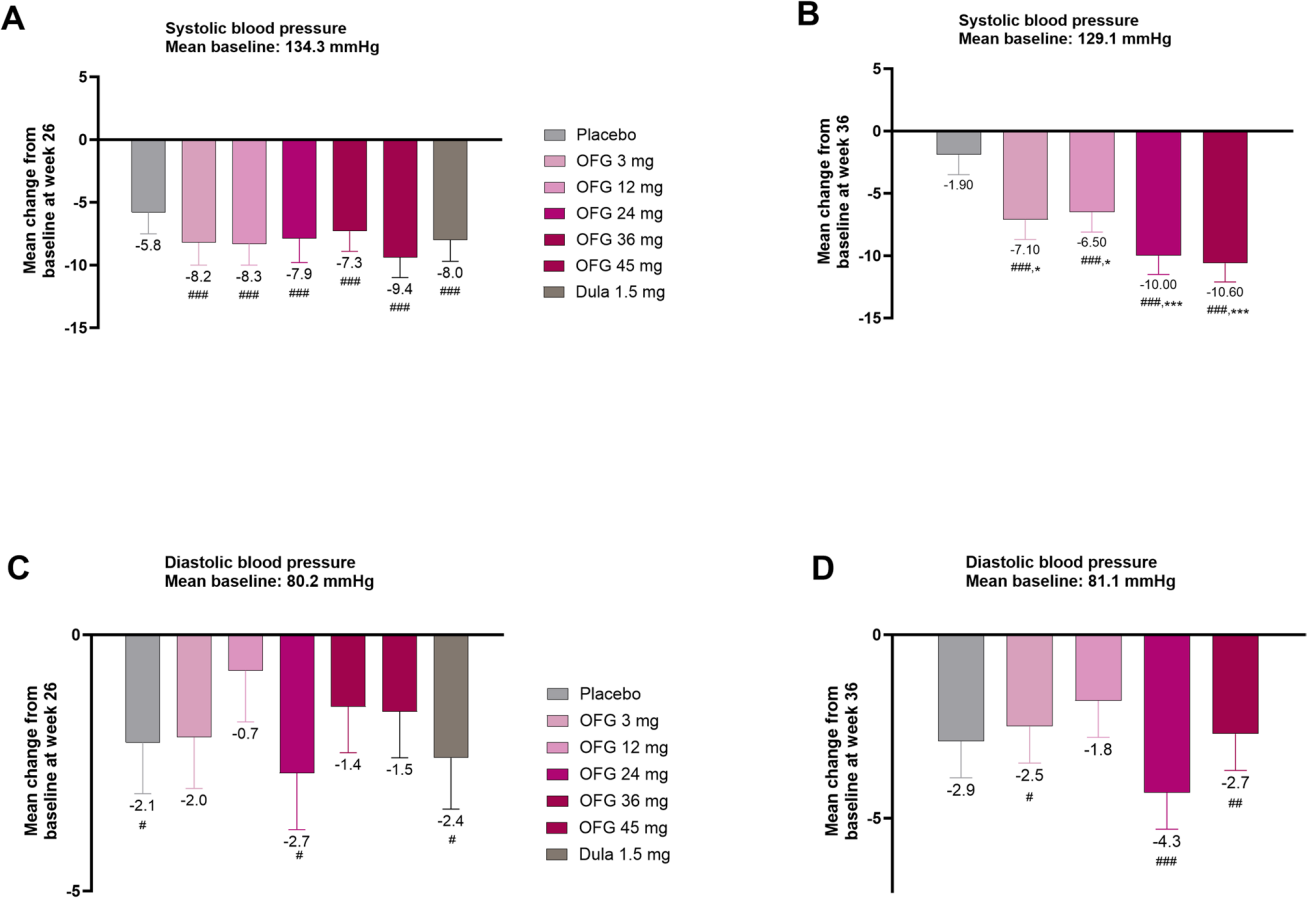
Treatment effects on blood pressure in participants with or without T2D. Data are LSM ± SE (baseline and change at week 26. #*p* < 0.05, ## *p* < 0.01, ### *p* < 0.001 change from baseline. *p < 0.05, **p < 0.01, ***p < 0.001 for OFG vs PBO. Mean change in **A** systolic blood pressure at week 26 in participants with T2D, **B** systolic blood pressure at week 36 in participants with obesity, **C** diastolic blood pressure at week 26 in participants with T2D **D** diastolic blood pressure at week 36 in participants with obesity

At week 26 of the T2D study, decreases in SBP were observed with orforglipron by − 7.3 to − 9.4 mmHg, compared to − 8.0 mmHg with dulaglutide and − 5.8 mmHg with placebo. The changes from baseline with orforglipron were significant, but they were not significantly different from placebo (Fig. 1; Supplemental Table 1). DBP decreased from baseline in all orforglipron treatment groups with decreases ranging from − 0.7 to − 2.7 mmHg, versus -2.4 mmHg with dulaglutide and − 2.1 mmHg with placebo (not significant for comparisons of orforglipron versus placebo; Fig. 1, Supplemental Table 1). Heart rate was increased with orforglipron (+ 2.6 to + 6.5 beats/min), with significantly greater increases at all doses compared to placebo (*p* < 0.01; Supplemental Table 1).

In the obesity trial, at week 36 SBP decreased from baseline with all orforglipron doses (− 6.5 to − 10.6 mmHg; Fig. 1, Supplemental Table 2), compared to -1.9 mmHg with placebo (*p* < 0.05; Fig. 1, Supplemental Table 2). Decreases in DBP with orforglipron (− 1.8 to − 4.3 mmHg) were significantly different from the − 2.9 mmHg change with placebo for the 12, 36, and 45 mg treatment groups (Fig. 1, Supplemental Table 2). In this population, heart rate was increased with orforglipron across all doses (+ 4.3 to + 7.9 beats/min), significantly different from baseline and from placebo (− 2.2 beats/min; Supplemental Table 2).

### Change from baseline in circulating lipids

**Fig. 2:**
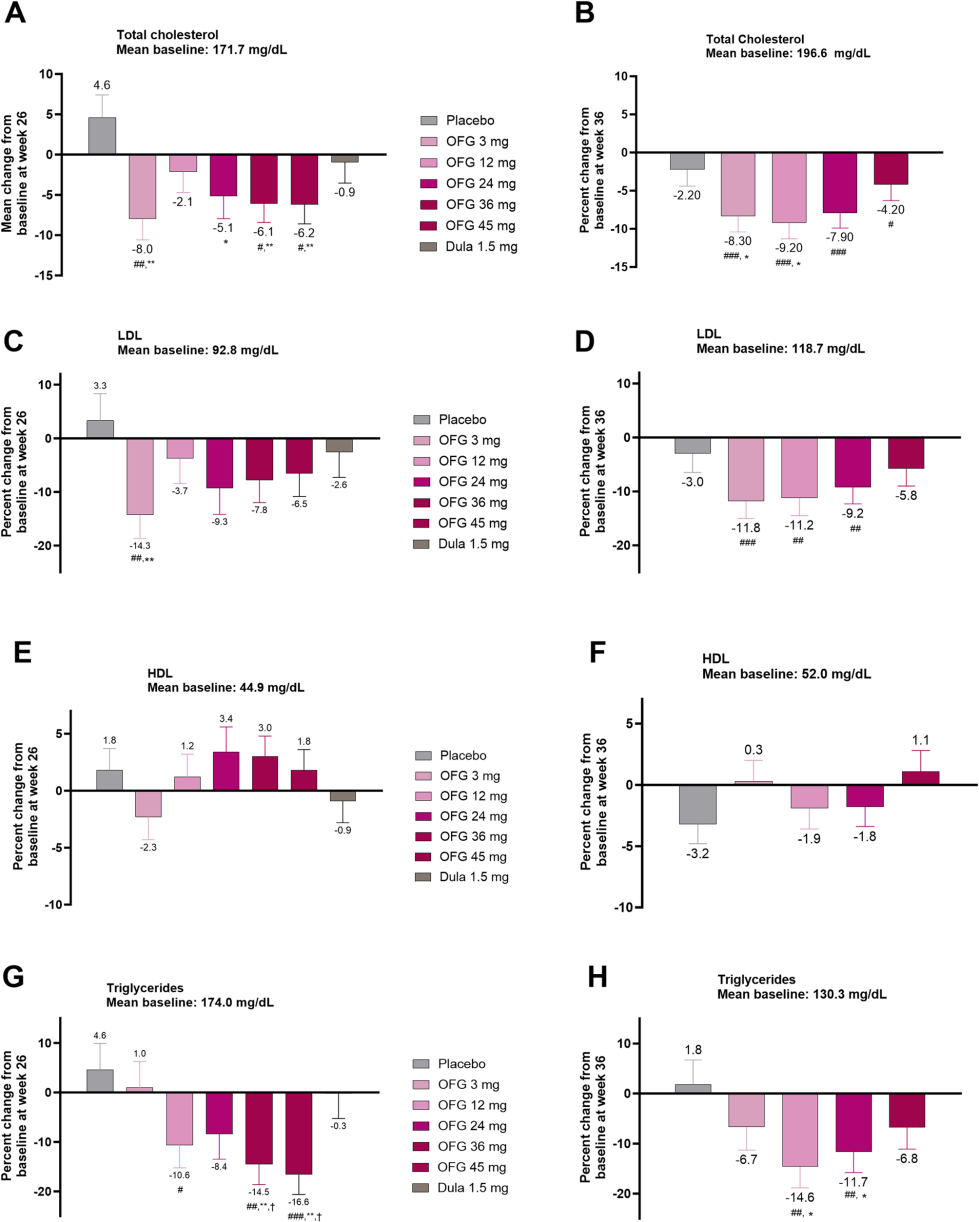
Treatment effects on lipid parameters in participants with or without T2D. Left (26-week T2D study), Right (36-week obesity study). Data are LSM ± SE (baseline and change at Week 26. #*p* < 0.05, ## *p* < 0.01, ### *p* < 0.001 change from baseline. *p < 0.05, **p < 0.01, ***p < 0.001 for OFG vs PBO. ^†^*p* < 0.05, ^††^*p* < 0.01, ^†††^*p* < 0.001 for OFG vs dulaglutide **A** Mean change in total cholesterol from baseline to week 26 in T2D. **B** Mean change in total cholesterol from baseline to week 36 in obesity. **C** Mean change in LDL from baseline to week 26 in T2D **D** Mean change in LDL from baseline to week 36 in obesity. **E** Mean change in HDL from baseline to week 26 in T2D **F** Mean change in LDL from baseline to week 36 in obesity. **G** Mean change in triglycerides from baseline to week 26 in T2D **H** Mean change in triglycerides from baseline to week 36 in obesity

Decreases in total cholesterol, LDL, very low-density lipoprotein (VLDL), and triglyceride were observed with all orforglipron doses in the T2D trial (Fig. 2; Supplemental Table 1). Following 26 weeks of treatment, the mean percent decrease observed in total cholesterol ranged from − 2.1 to − 8.0%, LDL cholesterol decreased from − 3.7 to − 14.3%, VLDL decreased from − 7.1 to − 16.4%, and triglyceride concentrations decreased by − 8.4 to − 16.6%. HDL cholesterol changes observed with orforglipron ranged from − 2.3 to + 3.4%, not different from baseline (Fig. 2, Supplemental Table 1). The observed decreases in total cholesterol were statistically different from placebo for 3, 24, 36, and 45 mg (*p* < 0.05; Table 3), and the decreases in VLDL were statistically different from placebo for 12, 36, and 45 mg (*p* < 0.05; Fig. 2, Supplemental Table 1).

Similar changes in circulating lipids were observed in the obesity study (Fig. 2, Supplemental Table 2). At 36 weeks, the mean percent decrease observed with orforglipron in total cholesterol ranged from − 4.2 to − 9.2%, LDL cholesterol decreased from − 5.8 to − 11.8%, VLDL cholesterol decreased from − 6.2 to − 14.4%, and triglyceride concentrations decreased from − 6.7 to − 14.6% (Fig. 2, Supplemental Table 2). HDL cholesterol changes associated with orforglipron varied from − 1.9 to + 1.1%, not different from baseline (Fig. 2, Supplemental Table 2). Compared to placebo, the decreases in total cholesterol were significant at 12 and 24 mg, while the decreases in triglyceride and VLDL were significant at 24 and 36 mg (*p* < 0.05; Supplemental Table 2).

### Change from baseline in apolipoproteins

**Fig. 3:**
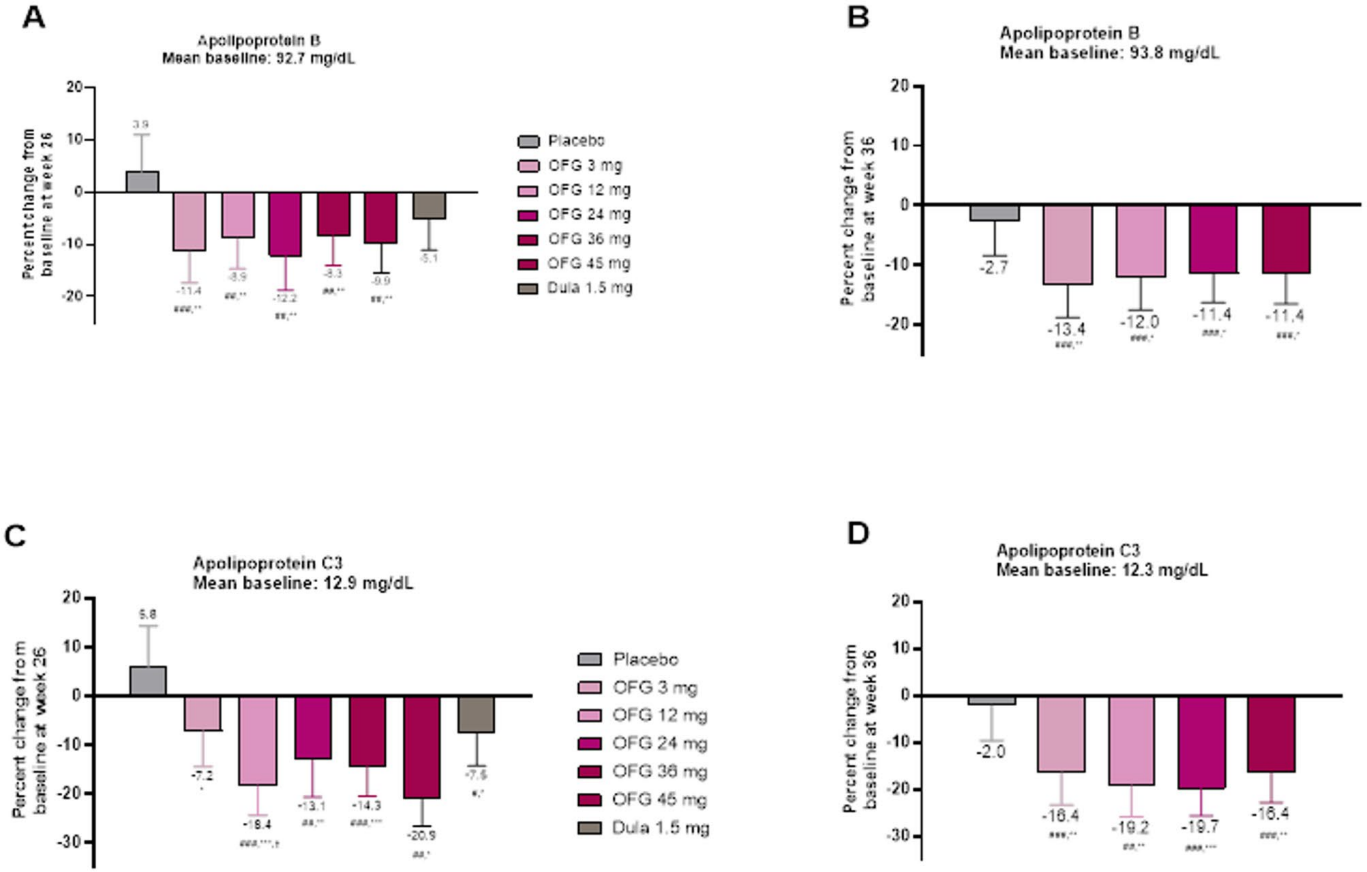
Treatment effects on apolipoproteins in participants with or without T2D. Data are LSM ± SE (baseline and change at week 26. #*p* < 0.05, ## *p* < 0.01, ### *p* < 0.001 change from baseline. *p < 0.05, **p < 0.01, ***p < 0.001 for OFG vs PBO. ^†^*p* < 0.05, ^††^*p* < 0.01, ^†††^*p* < 0.001 for OFG vs dulaglutide. **A** Mean change in ApoB from baseline to week 26 in T2D. **B** Mean change in ApoB from baseline to week 36 in obesity. **C** Mean change in ApoC3 from baseline to week 26 in T2D **D** Mean change in ApoC3 from baseline to week 36 in obesity

Significant reductions in ApoB concentrations from baseline were observed with orforglipron in the T2D trial (Fig. 3, Supplemental Table 1). Changes from baseline ranging from − 8.3 to − 12.2% were observed across all orforglipron doses. These decreases were statistically greater than placebo (+ 3.9%) at all doses but not different from dulaglutide (− 5.1%). Significant reductions from baseline were similarly observed for ApoC3 with all orforglipron doses, with reductions ranging from − 7.2 to − 20.9% (Fig. 1, Supplemental Table 1). These effects were statistically different from placebo (+ 5.8%) at all doses and compared to dulaglutide (− 7.5%) at 12 and 45 mg (Fig. 1, Supplemental Table 1).

In the obesity trial, at 36 weeks, ApoB decreases observed with orforglipron ranged from − 11.4 to − 13.4%, statistically different from the effect of placebo (− 2.7%; Fig. 3, Supplemental Table 2). ApoC3 decreases from baseline were also observed with orforglipron, with effects ranging from − 16.4 to − 19.7%, statistically different from placebo (− 2.0%; Fig. 3, Supplemental Table 2).

### Change from baseline in biomarkers of inflammation

**Fig. 4:**
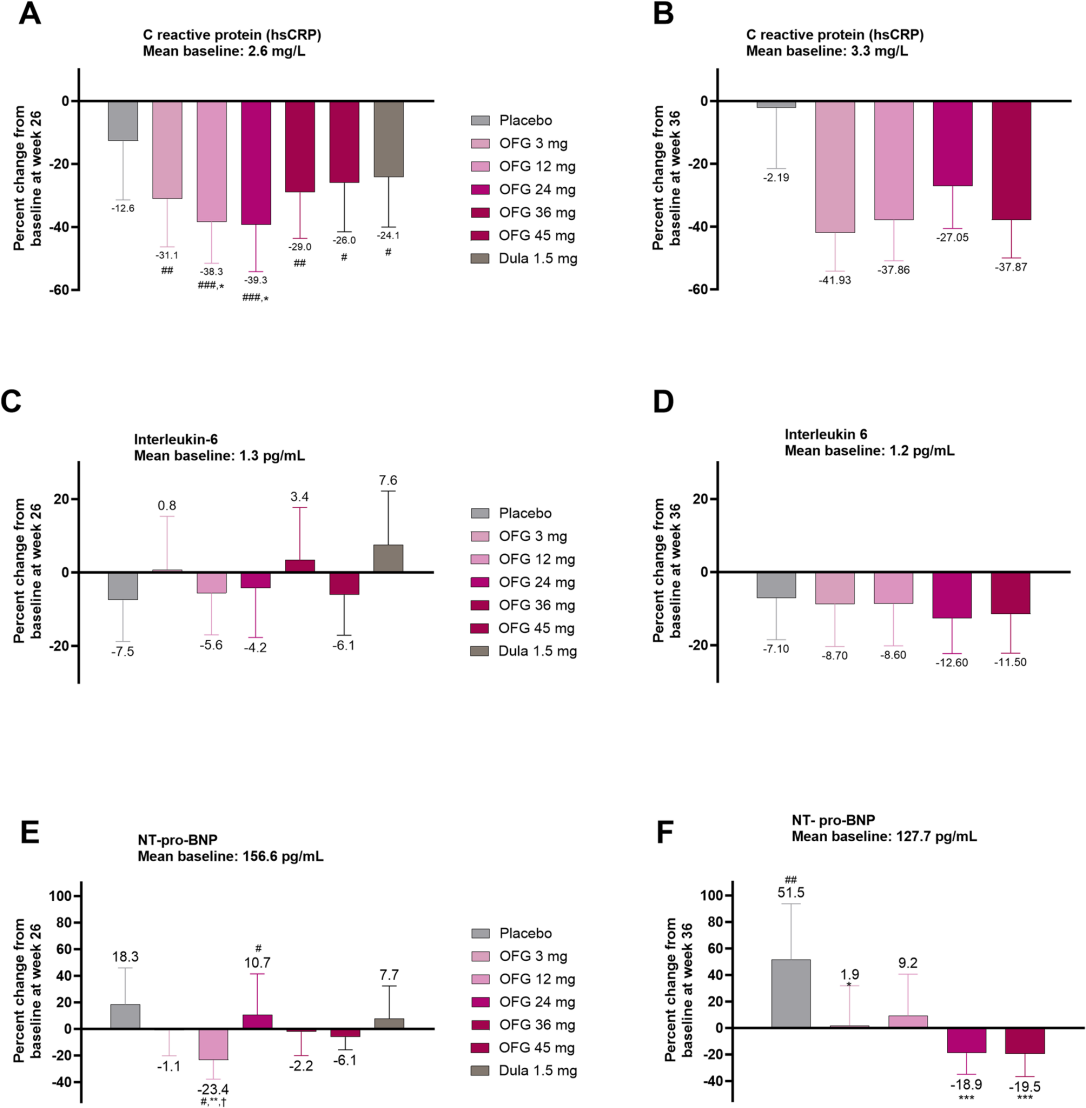
Treatment effects on hsCRP, IL-6 and NT-pro-BNP in participants with or without T2D. Data are LSM ± SE (baseline and change at week 26. #*p* < 0.05, ## *p* < 0.01, ### *p* < 0.001 change from baseline. *p < 0.05, **p < 0.01, ***p < 0.001 for OFG vs PBO. ^†^*p* < 0.05, ^††^*p* < 0.01, ^†††^*p* < 0.001 for OFG vs dulaglutide. **A** Mean change in high-sensitivity C reactive protein (hsCRP) at week 26 in T2D. **B** Mean change in high-sensitivity C reactive protein(hsCRP) at week 36 in obesity. **C** Mean change in Interleukin-6 at week 26 in T2D. **D** Mean change in Interleukin-6 at week 36 in obesity. (E)Mean change in NT-ProBNP at week 26 in T2D. **F** Mean change in NT-ProBNP at week 36 in obesity

In the T2D trial, significant reductions in hsCRP from baseline were observed with orforglipron with decreases ranging from − 26.0 to − 39.3%, statistically greater for 12 and 24 mg compared to placebo (− 12.6%) and dulaglutide (− 24.1%) (Fig. 4, Supplemental Table 1). IL-6 was not significantly changed from baseline with any dose of orforglipron (Fig. 4, Supplemental Table 1).

In the obesity trial, significant decreases in hsCRP were observed for all doses of orforglipron with effects ranging from − 27.1 to − 41.9%, statistically different from placebo (− 2.2%) at 12, 24, and 45 mg (Fig. 4, Supplemental Table 2). IL-6 changes ranged from − 8.6 to − 12.6% but only the effect of 36 mg orforglipron (− 12.6%) was statistically different from placebo (− 7.1%) (Fig. 4, Supplemental Table 2).

### Change from baseline in NT-pro-BNP

In the T2D trial changes in NT-pro-BNP were variable with orforglipron, ranging from − 23.4 to + 10.7% (Fig. 4, Supplemental Table 1). The change was + 18.3% with placebo and + 7.7% with dulaglutide. The effect observed with orforglipron 12 mg was statistically different from placebo (Fig. 4, Supplemental Table 1), but the overall pattern did not suggest a consistent treatment effect with orforglipron.

A variable pattern of response was also seen with orforglipron in the obesity trial, with NT-pro-BNP changes in individual treatment groups ranging from + 1.9 to − 19.5% (Fig. 4, Supplemental Table 2). In this trial, an apparent dose effect was observed, where higher doses of orforglipron were associated with larger decreases in NT-pro-BNP, with − 18.9% in the 36 mg group and − 19.5% in the 45 mg group, compared to a significant increase of + 51.5% with placebo (Fig. 4, Supplemental Table 2).

## Discussion

In this exploratory analysis using Phase 2 data from studies of participants with T2D or overweight/obesity without T2D, orforglipron treatment was associated with improvement in a number of traditional and novel markers of cardiovascular risk. Notable improvements included reductions of SBP, LDL cholesterol, and high-sensitivity C-reactive protein of a magnitude historically associated with cardiovascular benefit in studies of GLP-1 receptor agonists. CV risk factor improvements were seen at the mid-range doses of orforglipron, and higher doses did not result in further systematic reductions in these markers.

In many cardiovascular outcome trials (CVOT) with injectable GLP-1 RAs, the overall effect has been to reduce the rate of important cardiovascular disease outcomes including myocardial infarction, stroke, heart failure hospitalization, or death from cardiovascular disease [4, 5, 7, 27]. In addition to improvements in glycemia and weight, the treatment effects include improvement in traditional cardiovascular risk factors associated with the cardiovascular benefit and, in some cases, improvement in novel cardiovascular risk factors [9].

In the current analyses, placebo-adjusted reductions in SBP of up to − 1.5 mmHg and up to − 8.7 mmHg were observed with orforglipron in the obesity and T2D trials, respectively. In an exploratory analysis of CV risk factor changes in people with obesity but not T2D, aggregating data from STEP1 and STEP4 studies of the weight loss effects, a mean placebo-adjusted treatment effect of − 5.1 mmHg for SBP and − 2.4 mmHg for DPB was reported [17]. In a meta-analysis aggregating data from the full set of T2D registration trials, the average effect of semaglutide was to reduce SBP by 3.2 mmHg while no systematic reduction in DBP was observed [28]. In CVOTs with injectable GLP-1 RAs in people with T2D, the placebo-adjusted blood pressure reductions have been modest, ranging from − 0.7 to − 3.3 mmHg [4, 5, 7, 27, 29]. Also, in PIONEER 6, oral daily semaglutide up to 14 mg in participants with T2D produced a reduction in SBP of ~ 3 mmHg [8]; and in the SELECT trial using injected semaglutide in participants with overweight/obesity with pre-existing cardiovascular disease but without T2D, semaglutide 2.4 mg weekly produced a 3.3 mmHg reduction in SBP [6]. In the current analyses reductions from baseline in diastolic blood pressure were seen with orforglipron, but these changes were not different from placebo. A potential cardiovascular risk reduction associated with the blood pressure lowering effects of orforglipron may be inferred from the contributions of blood pressure lowering to the cardiovascular benefits of semaglutide and other GLP-1 receptor agonists.

In the current analyses, following treatment with orforglipron, we observed placebo-adjusted reductions in total cholesterol (up to − 12.0%), LDL cholesterol (up to − 17.0%), and triglycerides (up to − 20.3%). The association of reduction in cardiovascular risk with treatment-induced reductions in LDL cholesterol is recognized [30, 31], and the above studies of peptide-based GLP-1 RAs demonstrate reductions in total and LDL cholesterol concentrations in association with improved CV risk. Further, the magnitude of the effects of orforglipron described here is comparable to the effects of other GLP-1 RAs with demonstrated CV benefits. Contributions of treatment-induced reductions in triglyceride and increases in HDL cholesterol to reductions in CV risk are less directly demonstrated compared to the relationship with LDL cholesterol [18] but may signal improvements in fasting and meal-related lipid handling that favorably impact cardiovascular risk [32, 33].

Here we report significant reductions in Apolipoprotein B and Apolipoprotein C3 following treatment with orforglipron. These proteins correspond to lipoprotein particles that carry atherogenic cholesterol moieties and triglycerides respectively. Reductions in these apolipoproteins have been described in tandem with improvements in traditional lipid species following treatment with injected GLP-1RAs [32–36]. These changes may be prognostically important, as the relationships of cardiovascular risk with these apolipoproteins are additive to the risk associated with traditional lipid species [18].

Reductions in markers of systemic inflammation hsCRP (in both trials, 26–42%) and IL-6 (in the obesity trial, 4–13%) were also observed with orforglipron. Reductions in systemic inflammation are now generally accepted to confer cardiovascular benefit, as has been demonstrated in large-scale CVOTs with direct anti-inflammatory therapies. In a comprehensive meta-analysis, GLP-1 RAs were found to produce significant reductions in hsCRP [37]. In the SELECT cardiovascular outcome trial, semaglutide 2.4 mg in people with overweight and obesity without T2D produced a 38% reduction in hsCRP [6]. In the SUSTAIN and PIONEER trials, semaglutide delivered by injection and orally significantly reduced hsCRP levels in subjects with T2D, although the reduction was not statistically significant compared to active comparators in all trials [9]. The current observations of reduction in hsCRP with orforglipron suggest an anti-inflammatory effect, which may contribute to reductions in vascular outcomes.

The currently observed decreases in NT-pro-BNP following treatment with orforglipron (perhaps more convincingly in the obesity study, ~ 19% reduction at highest doses) further suggest a potential for cardiovascular benefit. Generally, changes in NT-pro-BNP are assessed in populations with cardiac ischemia or heart failure; such conditions were excluded from the current orforglipron trials. A post-hoc analysis of the REWIND trial, which also did not exclude such participants, found that lowering of NT-pro-BNP was associated with the beneficial MACE outcome [38]. In a pooled analysis of two heart failure studies in those with and without T2D, semaglutide 2.4 mg produced an 18% reduction in NT-pro-BNP, with similar magnitude of reduction across differing baseline weights [39]. A smaller study evaluating the effect of liraglutide on heart failure and natriuretic peptides in participants with and without T2D found a significant 25% reduction in NT-pro-BNP in participants with T2D but not in those without T2D [40]. These observations suggest that reductions in NT-pro-BNP with GLP-1 receptor agonist treatment may contribute to beneficial cardiovascular outcomes in those at risk.

The current analyses do not include formal comparisons of treatment effects between the obesity study and the T2D study. Broadly speaking, the improvements in the evaluated cardiovascular risk factors were similar between these study populations. It remains to be demonstrated whether the effect of orforglipron on cardiovascular outcomes will be similarly comparable between these populations.

Heart rates were increased by 2.4–7.9 bpm with orforglipron treatment in both study populations. This is expected for GLP-1 receptor agonists and has been observed in the studies that simultaneously demonstrate CV benefit. For example, in the REWIND trial dulaglutide at the commonly used dose of 1.5 mg in people with T2D was associated with an average increase of 1.9 bpm over 5 years of treatment exposure [4]. In the SELECT trial, the mean increase in heart rate after 2 years of treatment for people with obesity without T2D was 3.8 bpm [6]. Therefore, the current observed increase in heart rate is further evidence that orforglipron functions similarly to peptide-based GLP-1 receptors.

In most instances, we did not observe a strong relationship between the dose of orforglipron and the improvements in the various CV risk markers. This is somewhat in contrast with the observed dose response for weight and glycemia reductions in the primary results of the trials contributing data, although in the T2D trial in particular there was a convergence of efficacy outcomes across the uppermost doses [25, 26]. These observations may suggest that effects on biomarkers that reflect reductions in CV risk may not require exposure to the highest doses currently under evaluation.

A key strength of our study is the analysis of multiple biomarkers across various doses of orforglipron, providing a robust dataset for evaluating its effects on traditional and novel CV risk biomarkers. Additionally, the study populations included individuals without elevated baseline cardiovascular risk, suggesting these observations may be applicable to a broader population. The limitations of these analyses include their exploratory nature, as these studies were not specifically designed to assess changes in these markers. This is reflected in the relatively small sample sizes for each treatment group, which may have contributed to apparent inconsistencies in dose response. Consequently, the findings should be interpreted with caution, as they are not definitive and require further validation through larger, more targeted studies. Furthermore, while similar changes in CV risk factors have been linked to beneficial effects on CV outcomes with other agents, comparisons are complicated by the differences in the populations involved in those trials versus the current populations. Therefore, these comparisons should be interpreted with caution.

In conclusion, in these exploratory analyses, we observed beneficial effects of orforglipron treatment on traditional and novel cardiovascular risk factors, in people with T2D and in people with overweight/obesity without T2D. These effects are concordant with those observed with injectable peptide-based GLP-1 receptor agonists, including those where treatment effects to reduce cardiovascular disease events have been demonstrated.

## Electronic supplementary material

Below is the link to the electronic supplementary material.

Supplementary Material 1

## Data Availability

Lilly provides access to all individual participant data collected during the trial, after anonymisation, with the exception of pharmacokinetic data. Data are available to request 6 months after the indication studied has been approved in the US and EU and after primary publication acceptance, whichever is later. No expiration date of data requests is currently set once data are made available. Access is provided after a proposal has been approved by an independent review committee identified for this purpose and after receipt of a signed data sharing agreement. Data and documents, including the study protocol, statistical analysis plan, clinical study report, blank or annotated case report forms, will be provided in a secure data sharing environment. For details on submitting a request, see the instructions provided at www.vivli.org.

## Abbreviations

ApoB: Apolipoprotein B
ApoC3: Apolipoprotein C3
ANCOVA: Analysis of covariance
BMI: Body mass index
CI: Confidence interval
CV: Cardiovascular
CVD: Cardiovascular disease
CVOT: Cardiovascular outcome trials
DBP: Diastolic blood pressure
GLP-1RAs: Glucagon-like peptide 1 receptor agonists
HbA1c: Glycated haemoglobin
HDL: High-density lipoprotein
hsCRP: High-sensitivity C-reactive protein
IL-6: Interleukin-6
LC–MS/MS: Liquid chromatography- tandem mass spectrometry
LDL: Low-density lipoprotein
LSM: Least squares mean
MACE: Major adverse cardiovascular events
mITT: Modified intent-to-treat
MMRM: Mixed model repeated measures
NT-pro-BNP: N-terminal pro-type natriuretic peptide
N: Number of randomized participants
PBO: Placebo
OFG: Orforglipron
RCT: Randomized Clinical Trial
SBP: Systolic blood pressure
SD: Standard deviation
SE: Standard error
T2D: Type 2 Diabetes
VLDL: Very low-density lipoprotein
