# Supplementary material for: Treatment with orforglipron, an oral glucagon like peptide-1 receptor agonist, is associated with improvements of CV risk biomarkers in participants with type 2 diabetes or obesity without diabetes
Source: Cardiovasc Diabetol. 2025 Jun 6;24:240. doi: 10.1186/s12933-025-02781-x (PMC12142847; doi:10.1186/s12933-025-02781-x)
Supplement: Supplementary file 1 — Supplementary Material 1 [file 12933_2025_2781_MOESM1_ESM.docx]

**Supplemental appendix to Treatment with Orforglipron, an Oral Glucagon Like Peptide-1 Receptor Agonist, is Associated with Improvements of CV Risk Biomarkers in Participants with Type 2 Diabetes or Obesity Without Diabetes**

Sean Wharton^1^, Julio Rosenstock^2^, Manige Konige^3^, Yanzhu Lin^3^, Kevin Duffin^3^, Jonathan Wilson^3^, Hiya Banerjee^3^, Valentina Pirro^3^, Christof Kazda^3^, Kieren Mather^3^

**Author affiliations**

^1^McMaster University, York University, and Wharton Weight Management Clinic, Toronto, Canada

^2^Velocity Clinical Research at Medical City, Dallas, TX, USA

Eli Lilly and Company, Indianapolis, IN, USA

**Table of contents**

[**Supplemental Table 1: Treatment effects on cardiovascular risk factors in participants with T2D 35**](#_Toc197003246)

[**Supplemental Table 2: Treatment effects on cardiovascular risk factors in participants with obesity/overweight 40**](#_Toc197003247)

[**Supplemental Table 3: Concomitant drug use at baseline in patients with T2D 43**](#_Toc197003248)

[**Supplemental Table 4: Concomitant drug use at baseline in patients with obesity/overweight 47**](#_Toc197003249)

Supplemental Table 1: Treatment effects on cardiovascular risk factors in participants with T2D

|  | **PBO**  **(N=54)** | **Orforglipron** | | | | | **Dula 1.5 mg (N=50)** |
| --- | --- | --- | --- | --- | --- | --- | --- |
|  |  | **3 mg**  **(N=47)** | **12 mg**  **(N=50)** | **24 mg**  **(N=42)** | **36 mg**  **(N=59)** | **45 mg**  **(N=59)** |  |
| **Systolic blood pressure** | | | | | | | |
| Baseline (mmHg) | 135.1 (1.8) | 132.2 (1.9) | 135.5 (1.9) | 130.2 (2.0) | 132.9 (1.7) | 137.1 (1.7) | 135.7 (1.9) |
| Change from baseline at week 26 | -5.8 [-9.1, -2.6] | -8.2 [-11.6, -4.7]^###^ | -8.3 [-11.7, -5.0]^###^ | -7.9 [-11.6, -4.2]^###^ | -7.3 [-10.4, -4.2]^###^ | -9.4 [-12.5, -6.2]^###^ | -8.0 [-11.3, -4.7]^#^^##^ |
| Placebo-adjusted change from baseline [95%CI] | **-** | -2.3 [-7.1, 2.4] | -2.5 [-7.2, 2.2] | -2.1 [-7.0, 2.9] | -1.5 [-6.0, 3.0] | -3.5 [-8.0, 1.0] | -2.1 [-6.8, 2.5] |
| **Diastolic blood pressure** | | | | | | | |
| Baseline (mmHg) | 81.6 (1.1) | 77.9 (1.2) | 80.3 (1.2) | 78.8 (1.3) | 80.5 (1.1) | 80.5 (1.1) | 81.2 (1.2) |
| Change from baseline at week 26 | -2.1 [-4.1, -0.2] ^#^ | -2.0 [-4.1, 0.0] | -0.7 [-2.7, 1.2] | -2.7 [-4.8, -0.5]^#^ | -1.4 [-3.2, 0.4] | -1.5 [-3.3, 0.4] | -2.4 [-4.4, -0.5] ^#^ |
| Placebo-adjusted change from baseline [95%CI] | - | 0.1 [-2.7, 2.9] | 1.4 [-1.4, 4.2] | -0.5 [-3.4, 2.4] | 0.7 [-1.9, 3.4] | 0.7 [-2.0, 3.3] | -0.3 [-3.0, 2.5] |
| **Heart rate** |  |  |  |  |  |  |  |
| Baseline (beats/min) | 72.4 (1.3) | 72.1 (1.4) | 72.7 (1.4) | 73.1 (1.5) | 75.7 (1.3) | 75.7 (1.3) | 72.1 (1.4) |
| Change from baseline at week 26 | -1.9 [-4.3, 0.4] | 2.6 [0.2, 5.1]^#,**^ | 6.5 [4.1, 9.0]^###,***, †^ | 4.2 [1.6, 6.9]^##,***^ | 6.3 [4.0, 8.5]^###,***, †^ | 5.9 [3.7, 8.1]^###,***, †^ | 2.4 [0.0, 4.7]^*^ |
| Placebo-adjusted change from baseline [95%CI] | - | 4.6 [1.2, 8.0] | 8.5 [5.1,11.8] | 6.2 [2.6, 9.7] | 8.2 [5.0,11.5] | 7.8 [4.6,11.1] | 4.3 [0.9, 7.6] |
| **Total cholesterol** |  |  |  |  |  |  |  |
| Baseline (mg/dL) | 168.5 (5.4) | 164.0 (5.6) | 165.4 (5.5) | 156.8 (5.7) | 182.0 (5.6) | 168.6 (5.2) | 159.8 (5.3) |
| Change from baseline at week 26 | 7.7 [-1.4, 16.7] | -13.3 [-21.7, -4.9] | -3.5 [-12.2, 5.1] | -8.6 [-17.7, 0.5] | -10.2 [-17.9, -2.5] | -10.3 [-18.0, -2.6] | -1.5 [-10.2, 7.1] |
| % Change at week 26 | 4.6 [-0.8, 10.0] | -8.0 [-13.0, -3.0]^##,**^ | -2.1 [-7.3, 3.1] | -5.1 [-10.6, 0.3]^*^ | -6.1 [-10.7, -1.5]^#,**^ | -6.2 [-10.8, -1.6]^#,**^ | -0.9 [-6.1, 4.2] |
| Placebo-adjusted %change from baseline [95%CI] | - | -12.0 [-18.6, -5.4] | -6.4 [-13.3, 0.5] | -9.3 [-16.3, -2.3] | -10.2 [-16.6, -3.8] | -10.3 [16.7, -3.9] | -5.3 [-12.2, 1.7] |
| **LDL** |  |  |  |  |  |  |  |
| Baseline (mg/dL) | 86.7 (5.0) | 83.4 (5.2) | 84.6 (5.1) | 76.6 (5.0) | 96.9 (5.4) | 87.9 (4.8) | 80.5 (4.8) |
| Change from baseline at week 26 | 2.8 [-5.5, 11.2] | -12.2 [-19.5, -4.9] | -3.2 [-11.1, 4.7] | -7.9 [-16.1, 0.2] | -6.7 [-13.7, 0.4] | -5.6 [-12.8, 1.6] | -2.2 [-10.2, 5.7] |
| % Change at week 26 | 3.3 [-6.5, 13.1] | -14.3 [-22.8, -5.7]^##,**^ | -3.7 [-13.0, 5.5] | -9.3 [-18.8, 0.2] | -7.8 [-16.0, 0.4] | -6.5 [-14.9, 1.8] | -2.6 [-11.9, 6.7] |
| Placebo-adjusted %change from baseline [95%CI] | - | -17.0 [-28.4, -5.6] | -6.8 [-19.4, 5.8] | -12.2 [-24.6, 0.2] | -10.7 [-22.3, 0.9] | -9.5 [-21.3, 2.3] | -5.7 [-18.4, 6.9] |
| **VLDL** |  |  |  |  |  |  |  |
| Baseline (mg/dL) | 30.1 (1.9) | 30.8 (2.1) | 32.1 (2.1) | 30.9 (2.2) | 34.2 (2.1) | 28.6 (1.7) | 28.0 (1.8) |
| Change from baseline at week 26 | 1.7 [-1.4, 4.8] | 0.2 [-2.9, 3.4] | -3.3 [-5.9, -0.6] | -2.2 [-5.2, 0.9] | -3.9 [-6.3, -1.4] | -5.0 [-7.4, -2.7] | 0.0 [-3.0, 2.9] |
| % Change at week 26 | 5.5 [-4.7, 15.7] | 0.8 [-9.4, 11.0] | -10.6 [-19.4, -1.9]^#,*^ | -7.1 [-16.9, 2.8] | -12.6 [-20.5, -4.7] ^##,**,†^ | -16.4 (-24.0, -8.8) ^###,***,††^ | -0.1 [-9.7, 9.5] |
| Placebo-adjusted %change from baseline [95%CI] | - | -4.4 [-17.8, 8.9] | -15.3 [-26.9, -3.6] | -11.9 [-24.5, 0.8] | -17.2 [-28.1, -6.2] | -20.8 [-31.3, -10.2] | -5.3 (-18.2, 7.6] |
| **Triglyceride** |  |  |  |  |  |  |  |
| Baseline (mg/dL) | 150.8 (9.9) | 153.8 (10.8) | 163.8 (11.2) | 158.5 (11.0) | 175.2 (11.1) | 142.6 (9.0) | 142.3 (9.7) |
| Change from baseline at week 26 | 7.1 [-8.9, 23.1] | 1.5 [-14.7, 17.7] | -16.4 [-30.4, -2.5] | -13.0 [-28.5, 2.5] | -22.5 [-34.8, -10.2] | -25.7 [-37.8, -13.7] | -0.4 [-15.7, 14.8] |
| % Change at week 26 | 4.6 [-5.8, 14.9] | 1.0 [-9.5, 11.4] | -10.6 [-19.6, -1.6]^#,*^ | -8.4 [-18.4, 1.6] | -14.5 [-22.5, -6.6] ^##,**,†^ | -16.6 [-24.4, -8.8] ^###,**,†^ | -0.3 [-10.1, 9.6] |
| Placebo-adjusted %change from baseline [95%CI] | - | -3.5 [-17.3, 10.4] | -14.6 [-26.6, -2.5] | -12.4 [-25.3, 0.5] | -18.3 [-29.4, -7.2] | -20.3 [-31.2, -9.4] | -4.7 [-18.0, 8.7] |
| **HDL** | | | | | | | |
| Baseline (mg/dL) | 44.1 (1.5) | 41.8 (1.5) | 42.5 (1.5) | 41.7 (1.6) | 43.8 (1.5) | 44.9 (1.5) | 44.5 (1.6) |
| Change from baseline at week 26 | 0.8 [-0.9, 2.4] | -1.0 [-2.7, 0.7] | 0.5 [-1.1, 2.2] | 1.5 [-0.4, 3.3] | 1.3 [-0.3, 2.9] | 0.8 [-0.8, 2.3] | -0.4 [-2.0, 1.2] |
| % Change at week 26 | 1.8 [-2.0, 5.6] | -2.3 [-6.1, 1.5] | 1.2 [-2.6, 5.1] | 3.4 [-0.8, 7.7] | 3.0 [-0.6, 6.6] | 1.8 [-1.8, 5.4] | -0.9 [-4.6, 2.8] |
| Placebo-adjusted %change from baseline [95%CI] | - | -4.0 [-9.2, 1.2] | -0.6 [-5.9, 4.7] | 1.6 [-4.0, 7.2] | 1.2 [-4.0, 6.4] | 0.0 [-5.1, 5.1] | -2.7 [-7.8, 2.4] |
| **Apolipoprotein B** | | | | | | | |
| Baseline (mg/dL) | 93.7 (4.0) | 91.3 (4.3) | 91.1 (3.9) | 93.6 (4.8) | 99.7 (4.1) | 93.0 (3.8) | 89.9 (3.8) |
| % Change at week 26 | 3.9  [-2.7, 11.0] | -11.4^###,**^  [-17.4, -4.9] | -8.9^##,**^  [-14.7, -2.7] | -12.2^##,**^  [-18.8, -5.0] | -8.3^##,**^  [-14.0, -2.3] | -9.9^##,**^  [-15.5, -4.0] | -5.1  [-11.1, 1.3] |
| Placebo-adjusted %change from baseline [95%CI] |  | -14.8 [-22.6, -6.2] | -12.4 [-20.1, -3.9] | -15.5 [-23.7, -6.4] | −11.8 [−19.49, −3.4] | −13.3 [−20.9, -5.1] | −8.7 [−16.8, 0.11] |
| **Apolipoprotein C3** | | | | | | | |
| Baseline (mg/dL) | 12.9 (0.8) | 13.2 (0.9) | 13.1 (0.8) | 13.5 (1.0) | 14.7 (0.9) | 12.6 (0.7) | 12.0 (0.7) |
| % Change at week 26 | 5.8  [-2.0, 14.3] | -7.2^*^  [-14.5, 0.8]) | -18.4^###,***,†^  [-24.4,-11.9] | -13.1^##,**^  [-20.7,-4.8] | -14.3^###,***^  [-20.5,-7.6] | -20.9^###,***,††^  [-26.6,-14.8] | -7.5^#,*^  [-14.3, -0.1] |
| Placebo-adjusted %change from baseline [95%CI] | - | −12.3 [−21.5, −1.9] | −22.9 [−30.8, −14.1] | −17.9 [−27.1, −7.5] | −18.99 [−27.2, −9.9] | −25.2 [−32.8, −16.9] | −12.6 [−21.5, −2.6] |
| **High sensitivity C-reactive protein (hsCRP)** | | | | | | | |
| Baseline (mg/L) | 3.3 (0.5) | 2.2 (0.4) | 2.3 (0.4) | 2.5 (0.5) | 2.8 (0.4) | 2.0 (0.3) | 2.8 (0.4) |
| Baseline (mg/L) | 3.3 (0.5) | 2.2 (0.4) | 2.3 (0.4) | 2.5 (0.5) | 2.8 (0.4) | 2.0 (0.3) | 2.8 (0.4) |
| % Change at week 26 | -12.6  [-31.4, 11.4] | -31.1^##^  [-46.3, -11.7] | -38.3^###,*^  [-51.5, -21.5] | -39.3^###,*^  [-54.2, -19.6] | -29.0^##^  [-43.6, -10.6] | -26.0^#^  [-41.5, -6.5] | -24.1^#^  [-40.0, -4.0] |
| Placebo-adjusted %change from baseline [95%CI] | - | -21.2 [-44.2, 11.3] | -29.4 [-49.8, -0.9] | -30.6 [-52.1, 0.6] | -18.8 [-41.8, 13.2] | -15.4 [-39.5, 18.4] | -13.2 [-37.9, 21.4] |
| **Interleukin 6** | | | | | | | |
| Baseline (pg/mL) | 1.6 (0.1) | 1.1 (0.1) | 1.1 (0.1) | 1.4 (0.2) | 1.3 (0.1) | 1.2 (0.1) | 1.2 (0.1) |
| % Change at week 26 | -7.5  [-18.8, 5.3] | 0.8 [-11.8, 15.3] | -5.6  [-17.0, 7.4] | -4.2  [-17.7, 11.5] | 3.4  [-8.7, 17.1] | -6.1  [-17.1, 6.4] | 7.6  [-5.2, 22.2] |
| Placebo-adjusted %change from baseline [95%CI] | - | 9.0 [-9.5, 31.3] | 2.1 [-14.9, 22.6] | 3.6 [-15.1, 26.4] | 11.8 [-6.5, 33.7] | 1.6 [-15.1, 21.5] | 16.4 [-2.8, 39.5] |
| **NT-pro-BNP** | | | | | | | |
| Baseline (pg/mL) | 211.0 (32.1) | 150.0 (24.4) | 157.5 (24.1) | 144.4 (25.8) | 154.8 (22.8) | 149.9 (21.6) | 180.7 (27.1) |
| % Change at week 26 | 18.3  [-4.0, 45.9] | -1.1  [-20.3, 22.8] | -23.4^#,**,†^  [-37.8, -5.7] | 10.7^#^  [-13.4, 41.4] | -2.2  [-20.1, 19.8] | 3.1  [-15.7, 26.2] | 7.7  [-12.3, 32.3] |
| Placebo-adjusted %change from baseline [95%CI] | - | -16.4 [-38.1, 12.8] | -35.3 [-51.7, -13.2] | -6.5 [-32.2, 29.1] | -17.3 [-38.2, 10.5] | -12.84 [-34.7, 16.4] | -9.0 [-31.9, 21.8] |
| Data presented are LSM (SE) at the baseline and percent change from baseline at week 26 unless otherwise noted. #p<0.05, ##p<0.01, ###p<0.001 for change from baseline. *p<0.05, **p<0.01, ***p<0.001 for OFG vs PBO. † p<0.05, ††p<0.01, †††p<0.001 for OFG vs Dula. CI=confidence interval; Dula= dulaglutide; HDL= high-density lipoprotein; LDL= low-density lipoprotein; LSM= least squares mean; N= number of randomized participants in each treatment group; OFG= orforglipron; PBO= placebo; SE= standard error; T2D= type 2 diabetes; VLDL= very low-density lipoprotein cholesterol. | | | | | | | |

Supplemental Table 2: Treatment effects on cardiovascular risk factors in participants with obesity/overweight

|  | **PBO**  **(N=43)** | **Orforglipron** | | | |
| --- | --- | --- | --- | --- | --- |
|  |  | **12 mg**  **(N=42)** | **24 mg**  **(N=41)** | **36 mg**  **(N=47)** | **45 mg**  **(N=46)** |
| **Systolic blood pressure** | | | | | |
| Baseline (mmHg) | 128.5 (1.6) | 129.4 (1.7) | 129.6 (1.7) | 131.5 (1.6) | 126.9 (1.6) |
| Change from baseline at week 36 | -1.9 [-5.0, 1.2] | -7.1 [-10.3, -3.9]^###,*^ | -6.5 [-9.7, -3.3]^###,*^ | -10.0 [-13.0, -7.0]^###,***^ | -10.6 [-13.6, -7.6]^###,***^ |
| Placebo-adjusted change from baseline [95%CI] | - | -5.2 [-9.7, -0.7] | -4.5 [-9.0, -0.1] | -8.1 [-12.5, -3.8] | -8.7 [-13.0, -4.3] |
| **Diastolic blood pressure** | | | | | |
| Baseline (mmHg) | 80.9 (1.1) | 82.7 (1.2) | 81.7 (1.2) | 81.0 (1.1) | 79.5 (1.0) |
| Change from baseline at week 36 | -2.9 [-4.8, -1.0] | -2.5 [-4.5, -0.6]^#^ | -1.8 [-3.8, 0.2] | -4.3 [-6.2, -2.5]^###^ | -2.7 [-4.5, -0.8]^##^ |
| Placebo-adjusted change from baseline [95%CI] | - | 0.4 [-2.4,3.1] | 1.1 [-1.7,3.9] | -1.4 [-4.1,1.3] | 0.2 [-2.5,2.9] |
| **Heart rate** |  |  |  |  |  |
| Baseline (beats/min) | 69.4 (1.5) | 73.6 (1.5) | 72.6 (1.5) | 70.2 (1.4) | 70.5 (1.4) |
| Change from baseline at week 36 | -2.2 [-4.8, 0.4] | 5.3 [2.6, 8.0]^###,***^ | 4.3 [1.6, 7.0]^##,***^ | 7.9 [5.3, 10.4]^###,***^ | 6.9 [4.3, 9.4]^###,***^ |
| Placebo-adjusted change from baseline [95%CI] | - | 7.5 [3.7,11.3] | 6.5 [2.7,10.3] | 10.1 [6.4,13.8] | 9.1 [5.4,12.7] |
| **Total cholesterol** | | | | | |
| Baseline (mg/dL) | 197.4 (6.2) | 186.5 (6.1) | 199.4 (6.5) | 186.5 (5.7) | 192.0 (5.7) |
| Change from baseline at week 36 | -4.2 [-12.6, 4.1] | -15.9 [-23.9, -7.9] | -17.7 [-25.7, -9.7] | -15.2 [-22.7, -7.6] | -8.1 [-16.0, -0.3] |
| % Change at week 36 | -2.2 [-6.5, 2.1] | -8.3 [-12.4, -4.1]^###,*^ | -9.2 [-13.4, -5.1]^###,*^ | -7.9 [-11.8, -4.0]^###^ | -4.2 [-8.3, -0.1]^#^ |
| Placebo-adjusted % change from baseline [95%CI] | - | -6.2 [-12.2, -0.3] | -7.2 [-13.1, -1.3] | -5.8 [-11.6, 0.0] | -2.1 [-8.1, 3.9] |
| **LDL** | | | | | |
| Baseline (mg/dL) | 120.5 (5.6) | 112.2 (5.5) | 116.5 (5.8) | 105.0 (4.8) | 112.8 (5.0) |
| Change from baseline at week 36 | -3.4 [-11.1, 4.3] | -13.4 [-20.5, -6.2] | -12.6 [-19.9, -5.3] | -10.4 [-17.4, -3.5] | -6.6 [-13.7, 0.6] |
| % Change at week 26 | -3.0 [-9.8, 3.8] | -11.8 [-18.1, -5.5]^###^ | -11.2 [-17.6, -4.7]^##^ | -9.2 [-15.4, -3.1]^##^ | -5.8 [-12.1, 0.6] |
| Placebo-adjusted % change from baseline [95%CI] | - | -9.1 [-18.1, 0.0] | -8.4 [-17.6, 0.9] | -6.4 [-15.5, 2.7] | -2.9 [-12.3, 6.6] |
| **VLDL** | | | | | |
| Baseline (mg/dL) | 25.9 (1.7) | 20.8 (1.4) | 23.4 (1.6) | 23.4 (1.5) | 23.9 (1.5) |
| Change from baseline at week 36 | 0.5 [-1.8, 2.7] | -1.5 [-3.6, 0.7] | -3.4 [-5.3, -1.4] | -2.8 [-4.7, -0.9] | -1.6 [-3.6, 0.4] |
| % Change at week 26 | 2.1 [-7.5, 11.7] | -6.2 [-15.2, 2.8] | -14.4 [-22.7, -6.0]^##,*^ | -12.0 [-19.9, -4.0]^##,*^ | -6.9 [-15.3, 1.6] |
| Placebo-adjusted % change from baseline [95%CI] | - | -8.1 [-20.5, 4.3) | -16.1 [-27.5, -4.7] | -13.8 [-25.0, -2.5) | -8.8 [-20.7 ,3.2] |
| **Triglyceride** | | | | | |
| Baseline (mg/dL) | 130.0 (8.5) | 104.2 (7.1) | 117.1 (8.1) | 117.0 (7.5) | 119.5 (7.4) |
| Change from baseline at week 36 | 2.1 [-9.1, 13.4] | -7.9 [-18.4, 2.6] | -17.2 [-27.0, -7.4] | -13.7 [-23.1, -4.4] | -7.9 [-17.9, 2.0] |
| % Change at week 26 | 1.8 [-7.8, 11.4] | -6.7 [-15.6, 2.2] | -14.6 [-23.0, -6.3]^##,*^ | -11.7 [-19.6, -3.7]^##,*^ | -6.8 [-15.2, 1.7] |
| Placebo-adjusted % change from baseline [95%CI] | - | -8.4 [-20.7, 4.0) | -16.1 [-27.5, -4.8) | -13.2 [-24.5, -1.9) | -8.4 [-20.4, 3.6) |
| **HDL** | | | | | |
| Baseline (mg/dL) | 46.9 (1.8) | 49.2 (2.0) | 53.2 (2.2) | 51.2 (1.9) | 50.7 (1.9) |
| Change from baseline at week 36 | -1.6 [-3.2, 0.0] | 0.1 [-1.5, 1.8] | -0.9 [-2.6, 0.8] | -0.9 [-2.5, 0.7] | 0.5 [-1.1, 2.2] |
| % Change at week 26 | -3.2 [-6.5, 0.0] | 0.3 [-3.1, 3.7] | -1.9 [5.3, 1.5] | -1.8 [-4.9, 1.3] | 1.1 [-2.2, 4.3] |
| Placebo-adjusted % change from baseline [95%CI] | - | 3.7 [-1.2, 8.6) | 1.4 [-3.5, 6.3) | 1.5 [-3.2, 6.2) | 4.4 [-0.4, 9.3) |
| **Apolipoprotein B** | | | | | |
| Baseline (mg/dL) | 94.5 (1.3) | 92.8 (1.4) | 94.0 (1.4) | 94.5 (1.3) | 93.2 (1.3) |
| % Change at week 36 | -2.7  [-8.4, 3.4] | -13.4^###, **^  [-18.8, -7.6] | -12.0^###, *^  [-17.5, -6.2] | -11.4^###, *^  [-16.3, -6.1] | -11.4 ^###,*^  [-16.5, -5.9] |
| Placebo-adjusted %change from baseline [95%CI] | - | -11.0 [-18.5, -2.8] | -9.6 [-17.3, -1.2] | -9.0 [-16.3, -1.0] | -8.9 [-16.4, -0.8] |
| **Apolipoprotein C3** | | | | | |
| Baseline (mg/dL) | 12.8 (0.4) | 12.3 (0.4) | 11.6 (0.3) | 12.5 (0.3) | 12.3 (0.3) |
| % Change at week 36 | -2.0  [-9.6, 6.2] | -16.4^###,**^  [-23.2, -8.9] | -19.2^###,**^  [-25.8, -12.0] | -19.7^###,***^  [-25.6, -13.4] | -16.4^###,**^  [-22.8, -9.5] |
| Placebo-adjusted %change from baseline [95%CI] | - | -14.7 [-24.0, -4.1] | -17.5 [-26.8, -7.1] | -18.1 [-26.7, -8.4] | -14.7 [-23.9, -4.4] |
| **High sensitivity C-reactive protein (hsCRP**) | | | | | |
| Baseline (mg/L) | 3.5 (0.5) | 4.0 (0.6) | 3.0 (0.4) | 3.4 (0.4) | 2.8 (0.4) |
| % Change at week 36 | -2.2 [-21.5, 21.8] | -41.9^###,**^  [-54.2, -26.4] | -37.9 ^###,**^  [-50.9, -21.4] | -27.1^##^  [-40.6, -10.4] | -37.9 ^###,**^  [-50.0, -22.8] |
| Placebo-adjusted %change from baseline [95%CI] | - | -40.6 [-56.9, -18.3] | -36.5 [-54.1, -12.0] | -25.4 [-44.9, 1.0] | -36.5 [-53.5, -13.3] |
| **Interleukin 6** | | | | | |
| Baseline (pg/mL) | 1.1 (0.1) | 1.3 (0.1) | 1.3 (0.1) | 1.2 (0.1) | 1.2 (0.1) |
| % Change at week 36 | -7.1 [18.5, 6.0] | -8.7 [-20.4, 4.7] | -8.6 [-20.2, 4.6] | -12.6^#^ [-22.3, -1.5] | -11.5 [-22.2, 0.7] |
| Placebo-adjusted %change from baseline [95%CI] | - | -1.7 [-18.7, -18.7] | -1.7 [-18.7, 18.9] | -5.9 [-21.3, 12.5] | -4.7 [-20.8, 14.6] |
| **NT-pro-BNP** | | | | | |
| Baseline (pg/mL) | 119.5 (18.3) | 128.9 (20.2) | 141.1 (22.4) | 124.3 (17.8) | 120.5 (17.5) |
| % Change at week 36 | 51.5^##^ [18.5, 93.8] | 1.9^*^ [-21.3, 31.9] | 9.2 [-15.2, 40.6] | -18.9^***^ [-35.0, 1.3] | -19.5^***^ [-36.7, 2.3] |
| Placebo-adjusted %change from baseline [95%CI] | - | -32.8 [-52.8, -4.2] | -28.0 [-49.5, 2.8] | -46.5 [-61.7, -25.3] | -46.9 [-62.4, 25.0] |
| Data presented are LSM (SE) at the baseline and percent change from baseline at week 26 unless otherwise noted. #p<0.05, ##p<0.01, ###p<0.001 for change from baseline. *p<0.05, **p<0.01, ***p<0.001 for OFG vs PBO. CI= confidence interval; HDL= high-density lipoprotein; LDL= low-density lipoprotein; LSM= least squares mean; N= number of randomized participants in each treatment group;OFG= orforglipron; PBO= placebo; SE= standard error; VLDL= very low-density lipoprotein cholesterol. | | | | | |

Supplemental Table 3: Concomitant drug use at baseline in patients with T2D

|  | **Placebo**  **(N=54)** | **Orforglipron** | | | | | **Dulaglutide (N=50)** | **Total**  **(N=383)** |
| --- | --- | --- | --- | --- | --- | --- | --- | --- |
|  |  | **3 mg (N=47)** | **12 mg (N=50)** | **24 mg (N=42)** | **36 mg (N=59)** | **45 mg (N=59)** |  |  |
| **Subjects with baseline antihypertensive therapy** | 43 (78.2) | 34 (66.7) | 44 (78.6) | 39 (83.0) | 38 (62.3) | 49 (77.8) | 38 (76.0) | 285 (74.4) |
| **ACE inhibitors and CCB** | 1 (1.8) | 1 (2.0) | 2 (3.6) | 0 | 1 (1.6) | 1 (1.6) | 1 (2.0) | 7 (1.8) |
| Amlodipine; Perindopril | 1 (1.8) | 1 (2.0) | 1 (1.8) | 0 | 1 (1.6) | 1 (1.6) | 1 (2.0) | 6 (1.6) |
| Amlodipine; Ramipril | 0 | 0 | 1 (1.8) | 0 | 0 | 0 | 0 | 1 (0.3) |
| **ACE inhibitors and diuretics** | 0 | 0 | 3 (5.4) | 1 (2.1) | 3 (4.9) | 2 (3.2) | 1 (2.0) | 10 (2.6) |
| Indapamide; Perindopril | 0 | 0 | 2 (3.6) | 0 | 1 (1.6) | 2 (3.2) | 0 | 5 (1.3) |
| Hydrochlorothiazide; Lisinopril | 0 | 0 | 1 (1.8) | 1 (2.1) | 2 (3.3) | 0 | 0 | 4 (1.03) |
| Hydrochlorothiazide; Quinapril | 0 | 0 | 0 | 0 | 0 | 0 | 1 (2.0) | 1 (0.3) |
| **ACE inhibitors, other combinations** | 1 (1.8) | 1 (2.0) | 3 (5.4) | 2 (4.3) | 2 (3.3) | 2 (3.2) | 1 (2.0) | 12 (3.1) |
| Amlodipine; Indapamide; Perindopril | 1 (1.8) | 1 (2.0) | 2 (3.6) | 2 (4.3) | 2 (3.3) | 2 (3.2) | 1 (2.0) | 11 (2.9) |
| Bisoprolol; Perindopril | 0 | 0 | 1 (1.8) | 0 | 0 | 0 | 1 (0.3) | 1 (0.3) |
| **ACE inhibitors plain** | 21 (38.2) | 20 (39.2) | 22 (39.3) | 18 (38.3) | 18 (29.5) | 22 (34.9) | 19 (38.0) | 140 (36.6 |
| Lisinopril | 8 (14.5) | 6 (11.8) | 9 (16.1) | 8 (17.0) | 6 (9.8) | 10 (15.9) | 7 (14.0) | 54.1 (14.1) |
| Perindopril | 8 (14.5) | 6 (11.8) | 6 (10.7) | 5 (10.6) | 8 (13.1) | 8 (12.7) | 7 (14.0) | 48 (12.5) |
| Ramipril | 4 (7.3) | 6 (11.8) | 4 (7.1) | 4 (8.5) | 2 (3.3) | 4 (6.3) | 3 (6.0) | 27 (7.0) |
| Benazepril | 0 | 0 | 0 | 1 (2.1) | 1 (1.6) | 0 | 1 (2.0) | 3 (0.8) |
| Trandolapril | 0 | 0 | 2 (3.6) | 0 | 1 (1.6) | 0 | 0 | 3 (0.8) |
| Captopril | 0 | 0 | 0 | 0 | 0 | 1 (1.6) | 1 (2.0) | 2 (0.5) |
| Enalapril | 0 | 1 (2.0) | 1 (1.8) | 0 | 0 | 0 | 0 | 2 (0.5) |
| Quinapril | 1 (1.8) | 0 | 0 | 0 | 0 | 0 | 0 | 1 (0.3) |
| Zofenopril | 0 | 1 (2.0) | 0 | 0 | 0 | 0 | 0 | 1 (0.3) |
| **Aldosterone antagonists** | 2 (3.6) | 3 (5.9) | 0 | 2 (4.3) | 3 (4.9) | 2 (3.2) | 2 (4.0) | 14 (3.7) |
| Spironolactone | 2 (3.6) | 2 (3.9) | 0 | 2 (4.3) | 2 (3.3) | 2 (3.2) | 2 (4.0) | 12 (3.1) |
| Eplerenone | 0 | 1 (2.0) | 0 | 0 | 1 (1.6) | 0 | 0 | 2 (0.5) |
| **Carvedilol** | 3 (5.5) | 0 | 1 (1.8) | 1 (2.1) | 0 | 2 (3.2) | 2 (4.0) | 9 (2.3) |
| **Apha/adrenoreceptor antagonists** | 1 (1.8) | 3 (5.9) | 2 (3.6) | 0 | 0 | 4 (6.3) | 0 | 10 (2.6) |
| Doxazosin | 1 (1.8) | 1 (2.0) | 2 (3.6) | 0 | 0 | 2 (3.2) | 0 | 6 (1.6) |
| Urapidil | 1 (1.8) | 2 (3.9) | 0 | 0 | 0 | 2 (3.2) | 0 | 2 (0.5) |
| **Amlodipine; Olmesartan** | 0 | 0 | 0 | 0 | 1 (1.6) | 1 (1.6) | 0 | 2 (0.5) |
| **ARBs and diuretics** | 1 (1.8) | 1 (2.0) | 2 (3.6) | 2 (4.3) | 1 (1.6) | 1 (1.6) | 1 (2.0) | 9 (2.3) |
| Hydrochlorothiazide; Losartan | 1 (1.8) | 0 | 2 (3.6) | 2 (4.3) | 0 | 0 | 0 | 5 (1.3) |
| Hydrochlorothiazide; Valsartan | 0 | 1 (2.0) | 0 | 0 | 1 (1.6) | 1 (1.6) | 1 (2.0) | 1 (0.3) |
| **Amlodipine; Hydrochlorothiazide; Olmesartan** | 0 | 0 | 0 | 0 | 0 | 1 (1.6) | 0 | 1 (0.3) |
| **ARBS, plain** | 9 (16.4) | 9 (17.6) | 10 (17.9) | 9 (19.1) | 10 (16.4) | 14 (22.2) | 12 (24.0) | 73 (19.1) |
| Losartan | 3 (5.5) | 4 (7.8) | 4 (7.1) | 3 (6.4) | 2 (3.3) | 7 (11.1) | 6 (12.0) | 29 (7.6) |
| Valsartan | 4 (7.3) | 1 (2.0) | 3 (5.4) | 2 (4.3) | 3 (4.9) | 5 (7.9) | 2 (4.0) | 20 (5.2) |
| Telmisartan | 2 (3.6) | 2 (3.9) | 2 (3.6) | 2 (4.3) | 2 (3.3) | 1 (1.6) | 2 (4.0) | 13 (3.4) |
| Candesartan | 0 | 1 (2.0) | 1 (1.8) | 1 (2.1) | 0 | 0 | 1 (2.0) | 4 (1.0) |
| Irbesartan | 0 | 0 | 0 | 1 (2.1) | 2 (3.3) | 1 (1.6) | 0 | 4 (1.0) |
| Olmesartan | 0 | 1 (2.0) | 0 | 0 | 1 (1.6) | 0 | 1 (2.0) | 3 (0.8) |
| Diltiazem | 0 | 1 (2.0) | 0 | 1 (2.1) | 0 | 0 | 0 | 2 (0.5) |
| **Beta-blockers non-selective** | 1 (1.8) | 0 | 1 (1.8) | 1 (2.1) | 0 | 0 | 1 (2.0) | 4 (1.0) |
| Propranolol | 1 (1.8) | 0 | 0 | 1 (2.1) | 0 | 0 | 0 | 2 (0.5) |
| Timolol | 0 | 0 | 1 (1.8) | 0 | 0 | 0 | 1 (2.0) | 2 (0.5) |
| **Beta-blockers selective** | 17 (30.9) | 18 (35.3) | 17 (30.4) | 18 (38.3) | 16 (26.2) | 16 (25.4) | 16 (32.0) | 118 (30.8) |
| Bisoprolol | 10 (18.2) | 8 (15.7) | 5 (8.9) | 9 (19.1) | 6 (9.8) | 6 (9.5) | 6 (12.0) | 50 (13.1) |
| Nebivolol | 4 (7.3) | 5 (9.8) | 5 (8.9) | 2 (4.3) | 5 (8.2) | 4 (6.3) | 6 (12.0) | 31 (8.1) |
| Metoprolol | 2 (3.6) | 5 (9.8) | 3 (5.4) | 4 (8.5) | 3 (4.9) | 5 (7.9) | 2 (4.0) | 24 (6.3) |
| Betaxolol | 1 (1.8) | 0 | 2(3.6) | 3 (6.4) | 1 (1.6) | 1 (1.6) | 1 (2.0) | 9 (2.3) |
| Atenolol | 0 | 0 | 2(3.6) | 0 | 1 (1.6) | 0 | 1 (2.0) | 4 (1.0) |
| **CCBs and diuretics** | 0 | 0 | 0 | 0 | 1 (1.6) | 0 | 0 | 1 (0.3) |
| Amlodipine; Indapamide | 0 | 0 | 0 | 0 | 1 (1.6) | 0 | 0 | 1 (0.3) |
| **Dihydropyridine derivatives** | 13 (23.6) | 9 (17.6) | 14 (25.0) | 10 (21.3) | 11 (18.0) | 16 (25.4) | 11 (22.0) | 84 (21.9) |
| Amlodipine | 11 (20.0) | 7 (13.7) | 13 (23.2) | 7 (14.9) | 8 (13.1) | 15 (23.8) | 9 (18.0) | 70 (18.3) |
| Lercanidipine | 0 | 0 | 0 | 3 (6.4) | 2 (3.3) | 1 (1.6) | 0 | 6 (1.6) |
| Lacidipine | 2 (3.6) | 1 (2.0) | 1 (1.8) | 0 | 0 | 0 | 1 (2.0) | 5 (1.3) |
| Nitrendipine | 0 | 1 (2.0) | 0 | 0 | 1 (1.6) | 1 (1.6) | 1 (2.0) | 4 (1.0) |
| **Imidazoline receptor agonists** | 1 (1.8) | 3 (5.9) | 5 (8.9) | 1 (2.1) | 2 (3.3) | 2 (3.2) | 1 (2.0) | 15 (3.9) |
| Rilmenidine | 0 | 2 (3.9) | 1 (1.8) | 1 (2.1) | 2 (3.3) | 2 (3.2) | 1 (2.0) | 9 (2.3) |
| Moxonidine | 1 (1.8) | 1 (2.0) | 3 (5.4) | 0 | 0 | 0 | 0 | 5 (1.3) |
| Clonidine | 0 | 0 | 1 (1.8) | 0 | 0 | 0 | 0 | 1 (0.3) |
| **Amiloride; Hydrochlorothiazide** | 0 | 0 | 1 (1.8) | 0 | 0 | 0 | 0 | 1 (0.3) |
| **Potassium spacing agents** | 0 | 1 (2.0) | 1 (1.8) | 0 | 0 | 0 | 0 | 2 (0.5) |
| Amiloride | 0 | 0 | 1 (1.8) | 0 | 0 | 0 | 0 | 1 (0.3) |
| Triamterene | 0 | 1 (2.0) | 0 | 0 | 0 | 0 | 0 | 1 (0.3) |
| **Verapamil** | 0 | 0 | 1 (1.8) | 2 (4.3) | 0 | 0 | 0 | 3(0.8) |
| **Sulfonamides** | 10 (18.2) | 5 (9.8) | 10 (17.9) | 9 (19.1) | 11 (18.0) | 12 (19.0) | 8 (16.0) | 65 (17.0) |
| Indapamide | 9 (16.4) | 4 (7.8) | 8 (14.3) | 7 (14.9) | 8 (13.1) | 8 (12.7) | 5 (10.0) | 49 (12.8) |
| Furosemide | 2 (3.6) | 0 | 1 (1.8) | 1 (2.1) | 1 (1.6) | 4 (6.3) | 1 (2.0) |  |
| Torasemide | 0 | 1 (2.0) | 1 (1.8) | 4 (8.5) | 2 (3.3) | 1 (1.6) | 1 (2.0) | 6 (1.6) |
| Chlortalidone | 0 | 0 | 0 | 1 (2.1) | 0 | 1 (1.6) | 1 (2.0) | 3 (0.8) |
| **Hydrochlorothiazide** | 8(14.5) | 6(11.8) | 7(12.5) | 4(8.5) | 5(8.2) | 3 (4.8) | 1 (2.0) | 34 (8.9) |
| **Subjects with baseline lipid lowering therapy** | 28(50.9) | 31 (60.8) | 31 (55.4) | 34 (72.3) | 32 (52.5) | 45 (71.4) | 29 (58) | 230 (60.1) |
| **HMG-COA reductase inhibitors** | 25 (45.5) | 30 (58.8) | 30 (53.6) | 30 (63.8) | 28 (45.9) | 41 (65.1) | 26 (52) | 210 (54.8) |
| Atorvastatin | 11(20.0) | 20(39.2) | 17(30.4) | 14 (29.8) | 12 (19.7) | 23 (36.5) | 14 (28.0) | 111(29.0) |
| Rosuvastatin | 8(14.5) | 7(13.7) | 9(16.1) | 14 (29.8) | 11 (18.0) | 13 (20.6) | 6 (12.0) | 68 (17.8) |
| Simvastatin | 3(5.5) | 2(3.9) | 4(7.1) | 2 (4.3) | 4 (6.6) | 3 (4.8) | 5 (10.0) | 23 (6.0) |
| Pravastatin | 3(5.5) | 0 | 0 | 0 | 1 (1.6) | 1 (1.6) | 0 | 5 (1.3) |
| Lovastatin | 0 | 1(2.0) | 0 | 0 | 0 | 1 (1.6) | 1 (2.0) | 3 (0.8) |
| Pitavastatin | 0 | 0 | 0 | 0 | 0 | 1 (1.6) | 0 | 1 (0.3) |
| **Fibrates** | 3(5.5) | 2(3.9) | 5(8.9) | 4 (8.5) | 5 (8.2) | 5 (7.9) | 8 (16.0) | 32 (8.4) |
| Fenofibrate | 2(3.6) | 2(3.9) | 5(8.9) | 3 (6.4) | 4 (6.6) | 5 (7.9) | 8 (16.0) | 29 (7.6) |
| Gemfibrozil | 1(1.8) | 0 | 0 | 1 (2.1) | 1 (1.6) | 0 | 0 | 3 (0.8) |
| **Other modifying agents** | 4(7.3) | 0 | 4(7.1) | 2 (4.3) | 3 (4.9) | 2 (3.2) | 1 (2.0) | 16 (4.2) |
| Ezetimibe | 3(5.5) | 0 | 1(1.8) | 1 (2.1) | 2 (3.3) | 2 (3.2) | 1 (2.0) | 10 (2.6) |
| Fish oil | 0 | 0 | 2(3.6) | 1 (2.1) | 1 (1.6) | 0 | 1 (2.0) | 5 (1.3) |
| Evolocumab | 0 | 0 | 1(1.8) | 0 | 0 | 0 | 0 | 1(0.3) |
| Phospholipids | 1(1.8) | 0 | 0 | 0 | 0 | 0 | 0 | 1(0.3) |
| **Combination of various lipid modifying agents** | 1(1.8) | 0 | 0 | 0 | 1(1.6) | 1(1.6) | 0 | 3(0.8) |
| Ezetimibe; Rosuvastatin | 0 | 0 | 0 | 0 | 1(1.6) | 1(1.6) | 0 | 2(0.5) |
| Fenofibrate; Simvastatin | 1(1.8) | 0 | 0 | 0 | 0 | 0 | 0 | 1(0.3) |
| **Subjects with prior antihyperglycemic therapy** | 13(23.6) | 4(7.8) | 9(16.1) | 8(17.0) | 9(14.8) | 5(7.9) | 4(8.0) | 52(13.6) |
| Sulfonylureas | 5(9.1) | 1(2.0) | 2(3.6) | 3(6.4) | 3(4.9) | 3(4.8) | 3(6.0) | 20(5.2) |
| Gliclazide | 3(5.5) | 1(2.0) | 2(3.6) | 0 | 2(3.3) | 1(1.6) | 3(6.0) | 12(3.1) |
| Glimepiride | 1(1.8) | 0 | 0 | 3(6.4) | 0 | 2(3.2) | 0 | 6(1.6) |
| Glipizide | 1(1.8) | 0 | 0 | 0 | 1(1.6) | 0 | 0 | 2(0.5) |
| GLP-1 analogues | 6(10.9) | 2(3.9) | 0 | 0 | 1(1.6) | 2(3.2) | 1(2.0) | 12(3.1) |
| Dulaglutide | 4(7.3) | 1(2.0) | 0 | 0 | 1(1.6) | 0 | 1(2.0) | 7(1.8) |
| Liraglutide | 2(3.6) | 0 | 0 | 0 | 0 | 1(1.6) | 0 | 3(0.8) |
| Semaglutide | 1(1.8) | 1(2.0) | 0 | 0 | 0 | 1(1.6) | 0 | 3(0.8) |
| SGLT-2 inhibitors | 3(5.5) | 1(2.0) | 2(3.6) | 3(6.4) | 1(1.6) | 0 | 1(2.0) | 11(2.9) |
| Dapagliflozin | 2(3.6) | 0 | 1(1.8) | 1(2.1) | 1(1.6) | 0 | 0 | 5(1.3) |
| Canagliflozin | 1(1.8) | 0 | 1(1.8) | 1(2.1) | 0 | 0 | 1(2.0) | 4(1.0) |
| Empagliflozin | 0 | 1(2.0) | 0 | 1(2.1) | 0 | 0 | 0 | 2(0.5) |
| Biguanides | 2(3.6) | 0 | 1(1.8) | 2(4.3) | 2(3.3) | 1(1.6) | 0 | 8(2.1) |
| Metformin | 2(3.6) | 0 | 1(1.8) | 2(4.3) | 2(3.3) | 1(1.6) | 0 | 8(2.1) |
| DPP-4 inhibitors | 2(3.6) | 0 | 2(3.6) | 1(2.1) | 2(3.3) | 0 | 0 | 7(1.8) |
| Sitagliptin | 2(3.6) | 0 | 1(1.8) | 0 | 2(3.3) | 0 | 0 | 5(1.3) |
| Linagliptin | 0 | 0 | 1(1.8) | 1(2.1) | 0 | 0 | 0 | 2(0.5) |
| **Combination of oral blood glucose lowering drugs** | 0 | 0 | 3 (5.4) | 0 | 0 | 0 | 0 | 3(0.8) |
| Metformin; Litagliptin | 0 | 0 | 2(3.6) | 0 | 0 | 0 | 0 | 2(0.5) |
| Dapagliflozin; Metformin | 0 | 0 | 1(1.8) | 0 | 0 | 0 | 0 | 1 (0.3) |
| **Insulins and analogues** | 0 | 0 | 0 | 0 | 2(3.3) | 1(1.6) | 0 | 3(0.8) |
| Fast-acting insulin | 0 | 0 | 0 | 0 | 2(3.3) | 0 | 0 | 2(0.5) |
| Human insulin | 0 | 0 | 0 | 0 | 0 | 1(1.6) | 0 | 1 (0.3) |
| Long-acting insulin glargine | 0 | 0 | 1(1.8) | 0 | 0 | 0 | 0 | 1 (0.3) |
| Data reported is from main study population and are presented are n (%) therapy at baseline unless otherwise noted. | | | | | | | | |

Supplemental Table 4: Concomitant drug use at baseline in patients with obesity/overweight

|  | **Placebo**  **(N=47)** | **Orforglipron** | | | | **Total**  **(N= 272)** |
| --- | --- | --- | --- | --- | --- | --- |
|  |  | **12 mg**  **(N=43)** | **24 mg**  **(N=43)** | **36 mg**  **(N=49)** | **45 mg**  **(N=52)** |  |
| **Number of subjects with antihypertensive therapy** | 19 (38.0) | 13 (26.0) | 21 (39.6) | 28 (48.3) | 25 (41.0) | 106 (39.0) |
| **ACE inhibitors and diuretics** | 0 | 1 (2.0) | 0 | 2 (3.4) | 0 | 3 (1.1) |
| Hydrochlorothiazide; Lisinopril | 0 | 0 | 0 | 1 (1.7) | 0 | 1 (0.4) |
| Hydrochlorothiazide; Ramipril | 0 | 0 | 0 | 1 (1.7) | 0 | 1 (0.4) |
| Indapamine; Perindopril | 0 | 1 (2.0) | 0 | 0 | 0 | 1 (0.4) |
| **ACE inhibitors plain** | 8 (16.0) | 3 (6.0) | 8 (15.1) | 12 (20.7) | 8 (13.1) | 39 (14.3) |
| Lisinopril | 4 (8.0) | 2 (4.0) | 5 (9.4) | 5 (8.6) | 4 (6.6) | 20 (7.4) |
| Perindopril | 2 | 1 (2.0) | 3 (5.7) | 6 (10.3) | 0 | 12 (4.4) |
| Ramipril | 0 | 0 | 0 | 1 (1.7) | 2 (3.3) | 3 (1.1) |
| Enalapril | 1 (2.0) | 0 | 0 | 0 | 1 (1.6) | 2 (0.7) |
| Fosinopril | 1 (2.0) | 0 | 0 | 0 | 0 | 1 (0.4) |
| Quinapril | 0 | 0 | 0 | 0 | 1 (1.6) | 1 (0.4) |
| **Spironolactone** | 0 | 0 | 0 | 2 (3.4) | 0 | 2 (0.7) |
| **Carvedilol** | 0 | 0 | 0 | 2 (3.4) | 0 | 4 (1.5) |
| **Doxazosin** | 0 | 0 | 0 | 1 (1.7) | 0 | 1 (0.4) |
| **ARBS and diuretics** | 0 | 0 | 0 | 2 (3.4) | 3 (4.9) | 5 (1.8) |
| Hydrochlorothiazide;  Telmisartan | 0 | 0 | 0 | 1 (1.7) | 1 (1.6) | 2 (0.7) |
| Candesartan;  Hydrochlorothiazide | 0 | 0 | 0 | 0 | 1 (1.6) | 1 (0.4) |
| Hydrochlorothiazide;  Losartan | 0 | 0 | 0 | 0 | 1 (1.6) | 1 (0.4) |
| Hydrochlorothiazide;  Valsartan | 0 | 0 | 0 | 1 (1.7) | 0 | 1 (0.4) |
| **ARBS plain** | 6 (12.0) | 6 (12.0) | 8 (15.1) | 5 (8.5) | 9 (14.8) | 34 (12.5) |
| Losartan | 2 (4.0) | 2 (4.0) | 2 (3.8) | 2 (3.4) | 4 (6.6) | 12 (4.4) |
| Valsartan | 1 (2.0) | 1 (2.0) | 3 (5.7) | 1 (1.7) | 2 (3.3) | 8 (2.9) |
| Olmesartan | 2 (4.0) | 0 | 1 (1.9) | 0 | 1 (1.6) | 6 (2.2) |
| Telmisartan | 1 (2.0) | 0 | 1 (1.9) | 2 (3.4) | 0 | 4 (1.5) |
| Irbesartan | 0 | 0 | 1 (1.9) | 0 | 2 (3.3) | 3 (1.1) |
| Azilsartan | 0 | 1 (2.0) | 0 | 0 | 0 | 1 (0.4) |
| **Diltiazem** | 1 (2.0) | 0 | 0 | 0 | 1 (1.6) | 2 (0.7) |
| **Beta-blockers non-selective** | 0 | 0 | 1 (1.9) | 0 | 1 (1.6) | 2 (0.7) |
| Nadolol | 0 | 0 | 0 | 0 | 1 (1.6) | 1 (0.4) |
| propranolol | 0 | 0 | 1 (1.9) | 0 | 0 | 1 (0.4) |
| **Beta-blockers selective** | 3 (6.0) | 1 (2.0) | 4 (7.5) | 5 (8.6) | 7 (11.5) | 20 (7.4) |
| Metoprolol | 1 (2.0) | 1 (2.0) | 2 (3.8) | 2 (3.4) | 4 (1.5) | 10 (3.7) |
| Bisoprolol | 0 | 0 | 0 | 2 (3.4) | 2 (3.4) | 4 (1.5) |
| Nebivolol | 1 (2.0) | 0 | 1 (2.0) | 1 (2.0) | 1 (2.0) | 4 (1.5) |
| Atenolol | 1 (2.0) | 0 | 1 (2.0) | 0 | 0 | 2 (0.7) |
| **Dihydropyridine derivatives** | 6 (12.0) | 4 (8.0 | 5 (9.4) | 11 (19.0) | 5 (8.2) | 31 (11.4) |
| Amlodipine | 6 (12.0) | 4 (8.0 | 5 (9.4) | 11 (19.0) | 5 (8.2) | 29 (10.7) |
| Lercanidipine | 0 | 0 | 0 | 1 (1.7) | 1 (1.6) | 2 (0.7) |
| **Rilmenidine** | 0 | 0 | 0 | 0 | 1 (1.6) | 1 (0.4) |
| **Low ceiling diuretics and potassium sparing agents** | 2 (4.0) | 0 | 0 | 1 (1.7) | 0 | 3 (1.1) |
| Hydrochlorothiazide;  Triamterene | 1 (2.0) | 0 | 0 | 1 (1.7) | 0 | 2 (0.7) |
| Amiloride;  Hydrochlorothiazide | 1 (2.0) | 0 | 0 | 0 | 0 | 1 (0.4) |
| **Verapamil** | 0 | 0 | 1 (1.9) | 0 | 0 | 1 (0.4) |
| **Sulfonamides** | 1 (2.0) | 3 (6.0) | 2 (3.8) | 4 (6.9) | 0 | 10 (3.7) |
| Indapamide | 1 (2.0) | 1 (2.0) | 2 (3.8) | 3 (5.2) | 0 | 7 (2.6) |
| Chlortalidone | 0 | 1 (2.0) | 0 | 1 (1.7) | 0 | 2 (0.7) |
| Furosemide | 0 | 1 (2.0) | 0 | 0 | 0 | 1 (0.4) |
| **Hydrochlorothiazide** | 6 (12.0) | 3 (6.0) | 6 (11.3) | 5 (8.6) | 10 (16.4) | 30 (11.0) |
| Data reported is from main study population and are presented are n (%) therapy at baseline unless otherwise noted. | | | | | | |
